# Specification of Environmental Consequences of the Life Cycle of Selected Post-Production Waste of Wind Power Plants Blades

**DOI:** 10.3390/ma14174975

**Published:** 2021-08-31

**Authors:** Katarzyna Piotrowska, Izabela Piasecka

**Affiliations:** 1 Faculty of Mechanical Engineering, Lublin University of Technology, 20-618 Lublin, Poland; 2 Faculty of Mechanical Engineering, University of Science and Technology in Bydgoszcz, 85-796 Bydgoszcz, Poland; izabela.piasecka@utp.edu.pl

**Keywords:** CED, Eco-indicator 99, LCA, waste, wind energy, wind power plant blade, recycling

## Abstract

Wind power plants during generation of electricity emit almost no detrimental substances into the milieu. Nonetheless, the procedure of extraction of raw materials, production of elements and post-use management carry many negative environmental consequences. Wind power plant blades are mainly made of polymer materials, which cause a number of problems during post-use management. Controlling the system and the environment means such a transformation of their inputs in time that will ensure the achievement of the goal of this system or the state of the environment. Transformations of control of system and environment inputs, for example, blades production, are describing various models which in the research methodology, like LCA (Life Cycle Assessment), LCM (Life Cycle Management), LCI (Life Cycle Inventory), etc. require meticulous grouping and weighing of life cycle variables of polymer materials. The research hypothesis was assuming, in this paper, that the individual post-production waste of wind power plant blades is characterized by a different potential impact on the environment. For this reason, the aim of this publication is to conduct an ecological and energy life cycle analysis, evaluation, steering towards minimization and development (positive progress) of selected polymer waste produced during the manufacture of wind power plant blades. The analyzes were based on the LCA method. The subject of the research was eight types of waste (fiberglass mat, roving fabric, resin discs, distribution hoses, spiral hoses with resin, vacuum bag film, infusion materials residues and surplus mater), which are most often produced during the production of blades. Eco-indicator 99 and CED (Cumulative Energy Demand) were used as the computation procedures. The influence of the analyzed objects on human health, ecosystem quality and resources was appraised. Amidst the considered wastes, the highest level of depreciating impact on the milieu was found in the life cycle of resin discs (made of epoxy resin). The application of recycling processes would decrease the depreciating environmental influence in the context of the total life cycle of all analyzed waste. Based on the outcome of the analyzes, recommendations were proposed for the environmentally friendly post-use management of wind power plant blades, that can be used to develop new blade manufacturing techniques that better fit in with sustainable development and the closed-cycle economy.

## 1. Introduction

Human economic activity is based on the activities of using the natural resources of the circumambient nature, what in turn causes the introduction of contamination and waste arise from the refining of these resources into the environment. The surge in the portion of renewable energy sources in the global energy balances contributes to a more economical use of fossil energy resources, enhancing the condition of the environment by reducing the emission of pollutants to the atmosphere, water, soil and the amount of generated waste. The use of energy from renewable sources increases the level of energy security, creates new workplaces, promotes regional development and contributes to solving many ecological problems like, for example, reducing the greenhouse effect. Conventional energy is using huge amounts of coal, oil, gas and other non-renewable fuels where processes of their acquisition, processing and combustion make this sector of the economy characterized by the highest portion in the emission of hazardous substances to the environment (especially the atmospheric one) [1,2,3,4].

The use of renewable energy is always associated with a certain consumption of non-renewable resources because the materials necessary for the production of components, working units or entire devices are usually produced from fossil raw materials and with the use of energy from conventional sources. Even so, in the context of the whole life cycle (LC), renewable resources used to generate electricity significantly reduce the depletion of non-renewable resources [5,6,7].

Wind power plants (WPP) are classified as “environmentally friendly” energy sources, where key principles of sustainable development are met. This method of generating energy is related to lower CO_2_ emissions compared to conventional power plants. However, in the lifecycle of WPP, certain amounts of waste and harmful emissions are generated, mainly in the process of gathering resources, production and post-use management stages. Most of the load of WPP are plastics and materials that can be recycled without much difficulty, for example, steel, that is used in the production of tower or ductile iron, from which the hub is cast. Massively processed elements constitute from 85 to 90% of the weight of WPP. The recyclable elements constitute from 85 to 90% of the weight of WPP. The biggest problem, however, is with wind power plants blades (WPPB), as composite materials are used for their production. They have many advantages in use but are problematic in processing. While the weight of the blades is not too large compared to the entire weight of the WPP (one blade usually weighs more than 6–7 Mg), its size, volume and scale effect (continuous increase of energy in installed WPP) is causing the polymer waste control and development problem that becomes more and more noticeable. The problem with closing the cycle of products made of composites, however, is a multi-sector problem. The construction sector, the electric and electronic industry, transport and the maritime sector also have a problem with post-consumer polymer waste management [8,9,10,11,12].

Polymer plastics have changed people’s lives since they were invented in the 20th century, with many benefits on the one hand, but also enormous damage to the environment on the other. The biggest problem with them is that most of their types are extremely persistent and can take hundreds of years to decompose. There are many different methods of handling polymer waste, ranging from landfill and incineration to recycling and transformation into new products. However, all these processes have a greater or lesser impact on the environment, so choosing the best option for a particular material is not easy [13]. One of the available ways of comparing the potential environmental impact of various processes is Life Cycle Assessment. So far, various studies have been carried out and a number of LCA analyses have been carried out on the management of waste from polymers, for example, plastics for bottle production [14], municipal solid waste [15], plastic wastes in construction materials [16], plastic solid waste [17], electronic waste (e-waste) [18] or photovoltaic solar panel [19]. However, there are still no comprehensive LCA studies in the area of post-production waste, including post-production waste WPPB.

**Figure 1:**
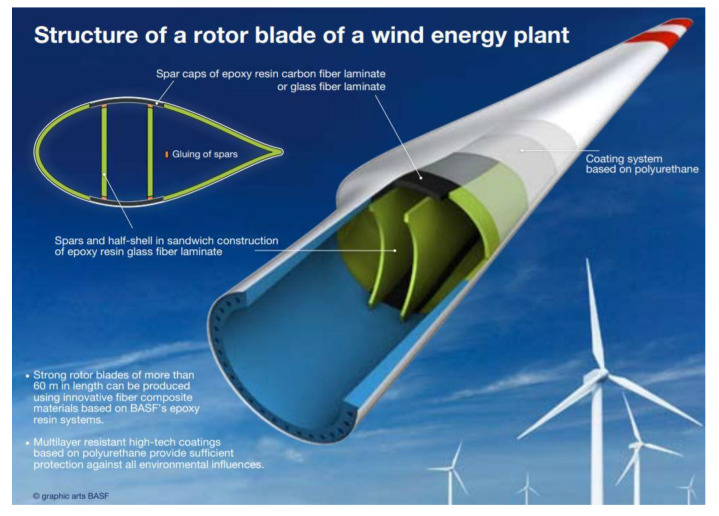
A cross section of the WPPB, including key materials and components [20].

The construction of an exemplary WPPB is shown in Figure 1.

Rationality is a feature of conscious human activity, consisting in selecting appropriate means to achieve the intended goals. There is a necessity to change the method we manage environmental resources and create more rational economy–environment relations. This process may take place on many levels, for example, through structural changes in sectors of the economy and changes in the production processes, operation and post-use management. An important application of ecological and energy analyzes is to assist the exploration for new solutions, design and develop production processes in such a way as to ensure the desired balance between economic and social development and respect for the environment and its protection. The fundamental tool used for this purpose is the Life Cycle Assessment (LCA) analysis, covering the entire LC of objects. By providing an assessment that takes into account both environmental and energy aspects, this analysis facilitates the design and selection of new ones, more environmentally friendly solutions, also in the field of WPPB production [21,22,23].

Life Cycle Assessment is a method that allows one to examine the entire LC of a test object (e.g., process, system and product), detecting their potential environmental impact. It is standardized (ISO 14040 and ISO 14044 standards) and covers all aspects of the impact of the object, from obtaining the raw material, through its production, operation and final post-use management. (i.e., cradle-to-grave) [24,25].

Thanks to the LCA method, it is possible to take into account almost all factors influencing the environment that are allied to production of waste of WPPB. During research, a structure is created within which it is easy to see and assess the relationships between the input and output elements (arising in each stage of the LC). The database prepared in such a way is the foundation for establishing the influence of the analyzed factors on the environment and enables the indication at which stage of the LC or within which process they carry the greatest potential risk. The wide scope of research results in the fact that the use of LCA gives tangible results, as it allows for a holistic look at both the analyzed object and the individual stages or processes in its LC. It specifies where certain threats to the environment arise [26,27].

The use of the LCA method is recommended in many current strategies and documents around the world, promoting the principles of sustainable development and activities directed at improving the ecological, energy and economic efficaciousness of processes or products. This is because it enables the comparison of various scenarios (strategies) and the selection of the most favorable variant, and on this basis, making decisions in the field of environmental policy. On the other hand, it is also used by manufacturers when designing new products or technologies and implementing life cycle management systems (LCM) [27,28].

Ecological and energy analyses are most often conducted in ordain to answer the question of what impact the LC of a given object, for instance, WPPB, may have on human health, the quality of the environment and the exhaustion of raw materials. LCA is a comprehensive way to estimate the interaction between the facility system and its surroundings. The complexity of this method results from its interdisciplinary nature. The implementation of the recommendations developed on the foundation of the results of the LCA analysis brings tangible benefits not only to enterprises, but also to the environment, and through this, to the entire society. It allows for the identification and awareness of the interdependence between human activity and its consequences for the environment. It also helps in the decision-making process aimed at minimizing the influence of industrial activities on the milieu, which is associated with the improvement of the environment. This is especially important now, when the key problems related to environmental pollution have already been identified, environmental awareness has increased, and the managements of a companies increasingly allocate more resources to activities related to the environmental protection than the minimum required by law. In the future, there is a possibility of creating a universal methodology for assessing the repercussions of economic activity on the milieu, based on the LCA method [27,29,30].

The main objective of this analysis was specification of environmental consequences of the LC of designated post-production waste of WPPB.

Despite the fact that this article is distinguished by a large volume, such a multifaceted subject as the ecological and energetic environment and system control examination of the LC of designated post-production waste of WPPB could not be completely explored in one work.

## 2. Models and Methods

### 2.1. Object and Plan of Control, Development Study

**Figure 2:**
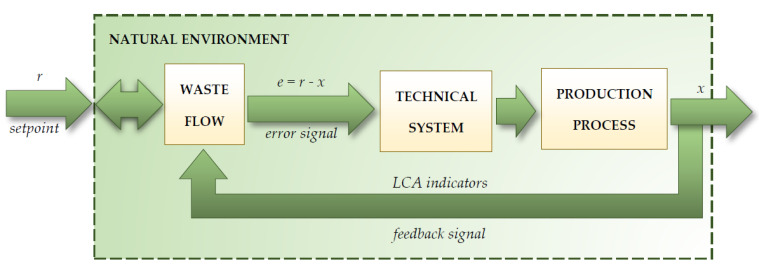
Flowchart of the closed arrangement of the milieu and WPP energy system: *r*—input of the system (blade production), *e*—error signal, *x*—output of the system (obtained products).

Eight types of post-production waste produced during the manufacture of WPPB, which were 49 m long, used in WPP with a capacity of 2 MW, were analyzed. These wastes included fiberglass mat, roving fabric, resin discs, distribution hoses, spiral hoses with resin, vacuum bag film, infusion materials residues and surplus materials. These were designated for research because of the highest mass fraction of all waste generated during the manufacture of blades. Additionally, these wastes generate many problems in post-use management as a result of the construction, composition and specificity of the materials they consist of (principally polymer materials). Supervising the system and the milieu is based on the transformation of their inputs in time that will enable the achievement of the purpose of a given system or the shape of the milieu. The system with one *r* input (materials) and one *x* output (obtained products) was analyzed. The functions *r*(*t*) and *x*(*t*) are defined for each *t*. Input *r* of the system (blade production) in Figure 2 is also the output of another system (environmental resources, system waste). If the latter system is a coalescence of the controller and an actuator, such as a control model, its input may be an error signal marked as *e*. The signal *e* is the deviation between the set value *r* and the system output value, then *x*. Control of the environment and the technical system is thus a kind of reactor with continuous composition. If the circulation of materials and waste is distressed, the overall response will be affected. The result will be environmental or technical system burden [31]. 

**Figure 3:**
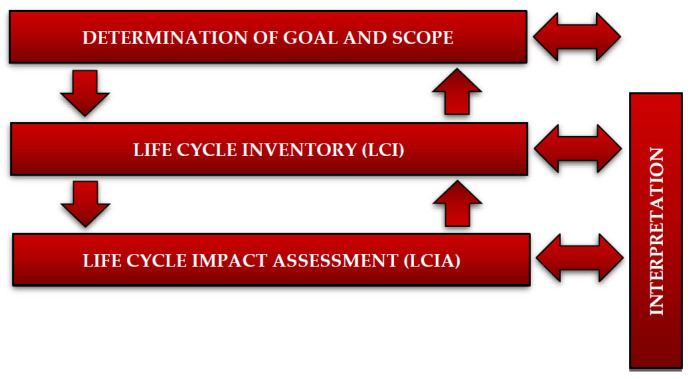
The stages of the LCA analysis (in accordance with ISO 14040 and 14044 standards).

The transformations of the system and environment inputs as well as their goals are described by various models, including groups of models and LCA (Life Cycle Assessment) methods or LCM (Life Cycle Management). For this reason, LCA was selected as the principal method to assess the potential consequences of WPPB post-production waste on human health, environmental quality and resource exhaustion. In accordance with ISO 14040 and ISO 14044 standards, the LCA analysis performed in this work included 4 phases: determination of goal and scope, life cycle inventory (LCI), life cycle impact assessment (LCIA) and interpretation (Figure 3) [32,33].

In the first step, the goal and scope of the research were composed (points are provided in Section 2.2). It resulted from an earlier audit of the latest state of knowledge and technology. It was noticed that the literature lacks exhaustive energy and milieu assessments of waste originated from the manufacture of WPPB. A key role in formulating the goal and scope was also played by collecting as much, as possible, the best quality data on the objects of analysis. This was achievable due to the combined effort with global companies producing WPPB. Not only samples of post-production waste were obtained, but also a complete set of data characterizing the blade production processes. A detailed description of the second stage of the research (LCI) is presented in Section 2.3. The next step included a comprehensive analysis of the LC of all the considered types of post-production waste. It covers a number of environmental aspects and an energy assessment. All the necessary simulations were performed using the SimaPro 8.4 software. The basic procedure used for the calculations was the Eco-indicator 99 method. It enabled the appraisal of the influence of processes occurring in the LC of post-production waste in three areas of influence: human health, ecosystem quality and resources. Additionally, it allowed to estimate the level of the harmful impact of emissions to the air, water and ground. The CED method was used to ascertain the size of the environmental influence of processes affiliated with acquiring energy in the LC of the considered waste. It allowed for a detailed analysis of the level of harmful impact on the environment of energy production processes, broken down into non-renewable and renewable sources. The exact course of this stage is presented in Section 2.4, and the obtained outcome and their discussion are presented in Section 3.1 and Section 3.2, respectively. The fourth and last stage (Section 2.5) included the exegesis of the obtained outcome. It is described in Section 3.1, Section 3.2 and Section 4 [34,35].

### 2.2. Determination of Goal and Scope

In Life Cycle Assessment, the scope of analysis contains functional unit, system boundaries, assumptions and limitations, data quality requirements and impact categories. The article analyzes the LC and the recycling processes of eight types of WPPB post-production waste. It was achievable to collect high-quality data on analyzed objects due to the combined effort with global WPPB companies. Specimens of post-production waste together with the numbers of data on blades manufacturing processes were attained. The performed research was directed at the implementation of the ecological and energetic milieu and system control scrutiny of the LC of designated postproduction waste of WPPB. The conducted research allowed to detail the existing reality (retrospective LCA) and shape future changes and define guidance for the implementation of greener, environmentally friendly solutions (prospective LCA). It was decided to carry out the standard Life Cycle Assessment process in line with the ISO 14040 and 14044 standards guidelines. The leading research assignment was to establish the extent of negative (or positive) influence of the LC of the assessed waste on human health, the quality of the environment and the exhaustion of natural resources. Additionally, an analysis of energy demand was performed, which was closely related to the above-mentioned task. A detailed assessment was carried out for fiberglass mat, roving fabric, resin discs, distribution hoses, spiral hoses with resin, vacuum bag film, infusion materials residues and surplus materials, which were the subjects of the research. The environmental aspects of the assessment include 11 impact categories specific to the Eco-indicator 99 model: carcinogens, resp. organics (organic compounds causing respiratory diseases), resp. inorganics (inorganic compounds causing respiratory diseases), climate change, radiation, ozone layer, ecotoxicity, acidification/eutrophication, land use, minerals and fossil fuels. The procured research conclusion was additionally grouped and compiled into three areas of influence: human health, ecosystem quality and resources. Four areas of emission of individual chemical compounds were also specified: air, water, soil and raw. The CED method was used for the energy review. The attained conclusion made it possible to ascertain the level of environmental influence of the processes affiliated to gaining energy in the LC and in the course of recycling of the considered post-production waste. They were divided into two main sources of energy: nonrenewable (fossil, nuclear) and renewable (biomass, wind, solar, geothe and water) [36,37,38,39].

The boundaries system in LCA must be defined in several dimensions. The key is, first of all, the delineation of the considered geographical area and the time horizon. The scope of the conducted research was related to European conditions because most of the processes taking place within the LC of research objects that transpire in Europe. That being the case, the scope of the audit was indicating to European conditions and geographical boundary system was acknowledged as territory of Europe. A precise time horizon has not been assumed. This was because the post-production waste of WPPB should be post-use management in the shortest possible period of time. A functional unit is a quantitative description of the product function, which is the basis for all calculations in the environmental impact assessment. A functional unit was clarified as the generation of 1 ton of a given type of waste (1 Mg). The audit did not include the stages of storing and transporting waste to places where it can undergo post-use management. The foremost reason was the divergent (but usually short) storage duration, as well as the significant gap in the outcome of delivery, which depend on the location of recycling plants or disposal area and the means of transport used for this purpose.

### 2.3. Life Cycle Inventory (LCI)

LCI is essentially about creating an input–output set. It is therefore a balance sheet analysis based on data inventory. Inputs and outputs were assigned to each unit process, and data related to its implementation were taken into account. Entrances included main materials, support materials and water requirement. The outputs, however, incorporate the primary product and emissions. In the scope related to the execution of processes, their time scale and media expenditure were distinguished. Abundance of the specifics was procured directly from the manufacturer of WPPB, and the remaining information was obtained from the SimaPro 8.4 software databases (Ecoinvent 3.4 database). In view of the confidentiality agreements concluded, details on the analyzed facilities and processed specifics have not been disclosed in this paper [40,41,42].

**Figure 4:**
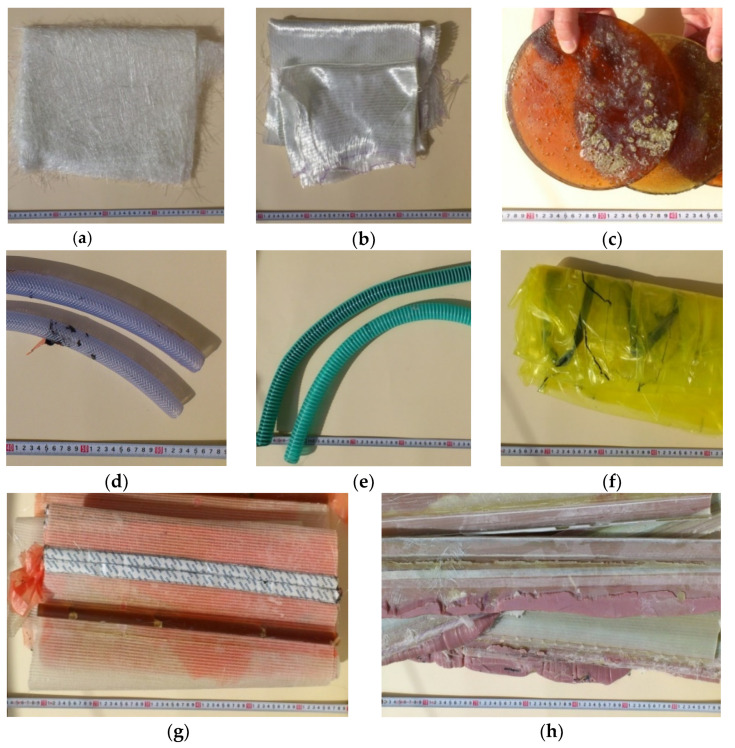
Investigated post-production waste of WPPB: (**a**) fiberglass mat, (**b**) roving fabric, (**c**) resin discs, (**d**) distribution hoses, (**e**) spiral hoses with resin, (**f**) vacuum bag film, (**g**) infusion materials residues, (**h**) surplus materials.

Eight types of waste were appraised: fiberglass mat (made of chopped glass fiber), roving fabric (made of epoxy silane-coated fiberglass), resin discs (made of epoxy resin), distribution hoses (inner and outer layer made of high quality PVC material, reinforcement—polyester fiber), spiral hoses with resin (wall made of high plasticized PVC, reinforcement—rigid PVC helix reinforced, inside—epoxy resin), vacuum bag film (made of co-extrusion of polyolefin and nylon based resins), infusion materials residues (epoxy resin, infusion mesh made of extruded LDPE grid mesh, release film made of highly flexible polypropylene-based release film, profile channel made of PVC, polyester release fabric made of polyester, flexible mold release tape made of polytetrafluoroethylene and silicone adhesive and sealant tape made of butyl) and surplus materials (polypropylene, epoxy resin, roving fabric made of epoxy silane-coated fiberglass, fiberglass mat made of chopped glass fiber, release film made of highly flexible polypropylene-based release film, polyester glue made of polyester fiberglass-reinforced resin). Figure 4 presents all the above-mentioned types of waste, while distribution hoses, spiral hoses with resin, vacuum bag film and infusion materials residues are waste auxiliary materials during the production of WPPB, which are not directly included in the material of the shovel but are necessary for its production [manufacturer’s data].

When all figures were assigned to unit processes, their validation was carried out, which was based on a bilateral energy and mass balance. Models were systematically formulated and filled with details. The magnitude of the inputs was equal to the size of the outputs. This procedure allowed for data aggregation, conversion into a functional unit and reference streams. As a result of summing up features of the same type (inputs of materials, energy, emissions, etc.) for individual unit processes, the input–output matrices were created. In the next step, those were assigned to reference streams, thanks to which inventory tables were created. All data had to be adapted to the SimaPro 8.4 software format. After introducing them to the program, it was possible to establish the 3rd phase of research—LCIA [35,43,44].

### 2.4. Life Cycle Impact Assessment (LCIA)

The analyzes under this study were carried out using the SimaPro 8.4 software (PRé Sustainability, LE Amersfoort, Netherlands) with Ecoinvent 3.4 database. The cut-off level adopted for the research was 0.1%. Ecological and energy review of the LC of designated WPPB was possible thanks to the availability of the two methods: Eco-indicator 99 and Cumulative Energy Demand. The results of the LCIA step are shown in Section 3.

#### 2.4.1. Eco-Indicator 99 Method

Eco-Indicator 99 is a tool for assessing the potential impact of the life cycle and was developed by PRé Consultants. Eco-indicator 99 allows to make an environmental assessment of the selected object (or objects) by calculating the numerical eco-indicator results for the materials and processes used. The obtained results indicate areas where a given object can be improved. Eco-indicator 99 is therefore, on the one hand, a scientific method of impact assessment and, on the other hand, a pragmatic eco-design method. It allows one to measure many different environmental impacts that are clearly presented in the final result as a single number [24,45,46].

**Figure 5:**
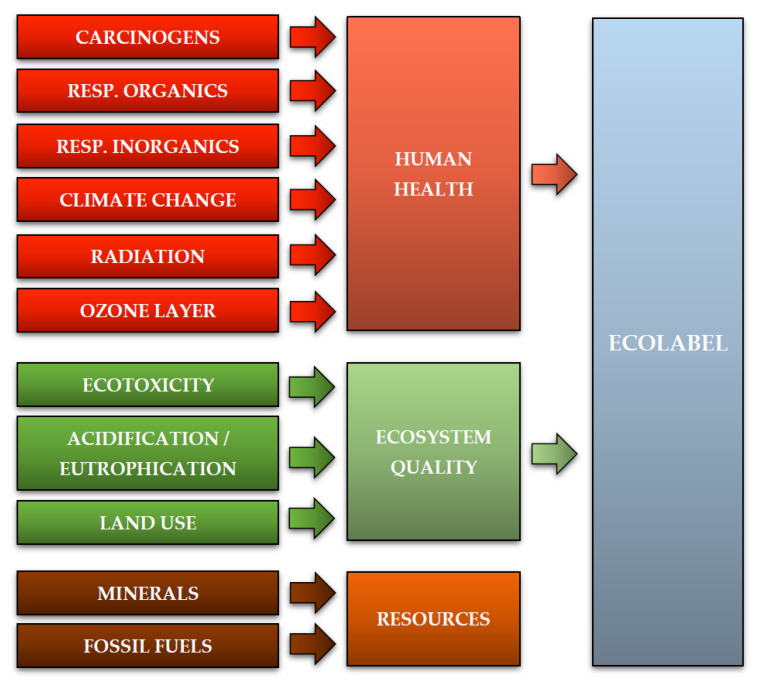
Method of data aggregation in the Eco-indicator 99.

The Eco-indicator 99 method is based on shaping the milieu influence at the endpoints of the environmental mechanism. As part of the analyzes using this model, all standard impact categories were taken into account: carcinogens, resp. organics, resp. inorganics, climate change, radiation, ozone layer, ecotoxicity, acidification/eutrophication, land use, minerals and fossil fuels. Their designation was accordant with the goal and scope of the scrutiny. The above-mentioned impact categories are arranged into 3 substantial collections/areas of influence: human health, ecosystem quality and resources. Areas of influence can be summed up in the form of a final Ecolabel after performing normalization, grouping and weighting (Figure 5) [42,45,46,47].

After the impact category had been established and selected, classification began. It involved assigning the LCI results to individual impact categories. Thanks to this procedure, it was possible to perform characterization, which includes calculating the value of the category index for the Life Cycle Impact results (using the characterization parameter). This enables the assessment of the degree of their participation in the amount related to a specified impact category. The outcome is the numerical estimate of the indicator. An example may be the impact category climate change, in which carbon dioxide and methane emissions play a key role. The sum of the result is the index value expressed as equivalent carbon dioxide, for example, kg CO_2_ eq. Similarly, the values of the indicator are determined for the remaining impact categories [35,48,49].

In the Eco-indicator 99 method, indicators are selected from a further level of the environmental mechanism. In the matter of human health, the DALY (Disability-Adjusted Life Years) unit was used, and the YLL (Years of Life Lost) and YLD (Years Lived Disabled) units were used as the category indicator. DALY is a global recognized unit applicable by WHO and the World Bank to appraise health statistics. Within its framework, countless diseases were assigned weights from 0 (perfect health) to 1 (death). Six impact categories are expressed in the DALY unit: carcinogens, resp. organics, resp. inorganics, climate change, radiation and ozone layer [35,50].

Impacts that reduce the quality ecosystem are very diverse. There is no common unit defined for this type of influence. In the Eco-indicator 99 method, the indicator is the species diversity level. The unit is PAF (Potentially Affected Fraction) or PDF (Potentially Disappeared Fraction). Within the 3 impact categories assessed in this area (ecotoxicity, acidification/eutrophication and land use), representative groups of species were adopted. For ecotoxicity (PAF·m^2^/yr), those are lower species of land and aquatic animals, while acidification/eutrophication and land use (PDF·m^2^/yr) were related to selected species of vascular plants [35,51].

A special damage indicator has been developed for resources: surplus energy expressed in MJ. The potential effects of a given extraction are the decrease in numbers of the functional components in the retainer or its total depletion. As a result of the reduction in supply (depletion or depletion of the deposit), it will be necessary to provide additional energy in order to extract the resource in question in the upcoming years or decades. Therefore, if the quality of a given resource decreases (as a result of its increased extraction), the labor to gain it from other sources escalate accordingly. The use of one kilogram of a given resource, on the one hand, is associated with a reduction in its quality, and on the other, with an increase in efforts to extract it (surplus energy). In Eco-indicator 99, the two impact categories are expressed in MJ surplus energy: minerals and fossil fuels [35,52,53].

The value of the category indicator (the size of the impact determined for a given impact category at the characterization stage) can be normalized. Normalization is the estimation of the value of a category indicator against a certain source value. In general, it therefore consists of dividing the obtained index value by the reference value. Total or average impacts for the sites in question are taken as reference values, for example, average annual impact per European resident. By means of using normalization, the share of a given effect in the total effect is determined. In this way, a standardized environmental profile is obtained. The obtained results are dimensionless and constitute the basis of, for instance, weighting. Normalization is a step that illustrates which effects are more or less represented in the final calculation result [42,53,54].

Grouping consists in assigning impact categories to one or more collections, in accordance with the goal and scope of the scrutiny. It includes organizing and, if possible, ranking of impact categories. During the LCIA, the divergent values of the impact category index can be weighted and totaled to achieve the weighting of the environmental repercussions. This allows, for example, to determine the multitude of detrimental influence of the greenhouse effect on the environment than acidification or eutrophication. Weighting involves designating weight respectively to impact category in a way the categories can be compared one to another. The most consequential impacts are most serious ones and are initial. By developing various models for aggregating LCA results into a single value, it was possible to carry out the weighting process based on an impact table based on numerical values. In the Eco-indicator 99 method, carrying out the weighting process produces environmental points (Pt) results. One-thousand environmental points are equivalent of milieu influence of 1 European in 1 year [35]. 

#### 2.4.2. CED Method

The Eco-indicator 99 method was employed to carry out ecological research, while the CED method was employed to shape the size of the environmental influence of the processes associated to procuring energy in the LC of WPPB. The CED (Cumulative Energy Demand) method allows you to calculate the cumulative energy demand. The impact indicators are split into 7 impact categories: 2 nonrenewable (fossil, nuclear) and 5 renewables (biomass, wind, solar, geothe, water). The obtained results are presented in MJ eq per 1 Mg (waste) [55,56,57].

### 2.5. Interpretation

The completeness check of the analysis was completed with a positive result. Every figure needed for the evaluation was concluded. These figures were acquired from a company manufacturing WPPB and from SimaPro 8.4 databases (Ecoinvent 3.4 database). A compliance check was also carried out through the assessment. The accepted assumptions, chosen methods, the depth of the analysis, and its itemized and precise inputs for the whole of the inspected post-production waste of WPPB were accordant with the purpose and range of the indagation. The obtained outcome of the analyzes for 8 types of waste and their evaluation are submit in Section 3 and Section 4 [35,42]. 

## 3. Results

The results of the examination executed as segment of the Life Cycle Impact Assessment (LCIA) are outlined in two sections: Eco-indicator 99 (Section 3.1) and CED (Section 3.2). The results of modeling with the use of Eco-indicator 99 were split up into 2 groups: results in the field of impact categories (Section 3.1.1) and areas of influence (Section 3.1.2). In addition, special attention was paid to the areas of emission of individual chemical compounds (compartments: air, water, soil and raw).

Total outcome is submitted as Pt per 1 Mg unit; it means the quantity of environmental points per 1 ton of the probed waste coming from the WPPB manufacturing activity [35,58].

### 3.1. Eco-Indicator 99

The first step in the area of examination operating Eco-indicator 99 was a comprehensive search of the 11 impact categories available under this method (carcinogens, resp. organics, resp. inorganics, climate change, radiation, ozone layer, ecotoxicity, acidification/eutrophication, land use, minerals and fossil fuels). 

In the second step, the obtained results were grouped into three basic areas of influence (human health, ecosystem quality and resources) and additionally analyzed. 

The conclusions were summarized separately for the LC and for the recycling processes of eight designated wastes from the production of WPPB (fiberglass mat, roving fabric, resin discs, distribution hoses, spiral hoses with resin, vacuum bag film, infusion materials residues and surplus materials) which are further characterized in Section 2.3.

#### 3.1.1. Impact Categories

When starting the analyzes under the impact categories, special attention was paid to estimate which of the 11 categories under consideration may be the origin of the greatest unit of obstructive (or constructive) milieu repercussions in the LC and recycling processes of selected post-production waste WPPB. 

It was established that the elevated volume of prospective detrimental outcome on the environment, in the case of all the studied objects, is characteristic of two impact categories: activity associated with the extraction of fossil fuels (from 812.31 to 62.69 Pt/1 Mg) and inorganic compounds causing respiratory diseases (from 94.75 to 30.56 Pt/1 Mg). It is the ramification of an excess demand for energy in the manufacture processes of WPPB and the directly related, extremely energy-consuming processes of extracting non-renewable raw materials, mandatory in individual processes in the course of the manufacture of blades. During the production of WPPB, many inorganic substances are also released into the environment, which are the cause of respiratory diseases, such as nitrogen oxides, sulfur dioxide or particulates (being a carrier, among others, for toxic chemical compounds, heavy metals, etc.) (Table 1).

On the other hand, the use of recycling processes for waste induced during the manufacture of WPPB may have many potential environmental benefits, especially in the two above-mentioned impact categories (fossil fuels and resp. inorganics). Recycling of the post-production waste in question allows for obtaining plastics and materials that can be used in other branches of the economy, which limits the consumption of matter and energy in a given production process to which they go. In addition, it is also associated with the reduction of harmful emissions to the atmospheric, water and/or soil environment and the minimization of the generation of further waste (Table 1 and Table 2).

**Figure 6:**
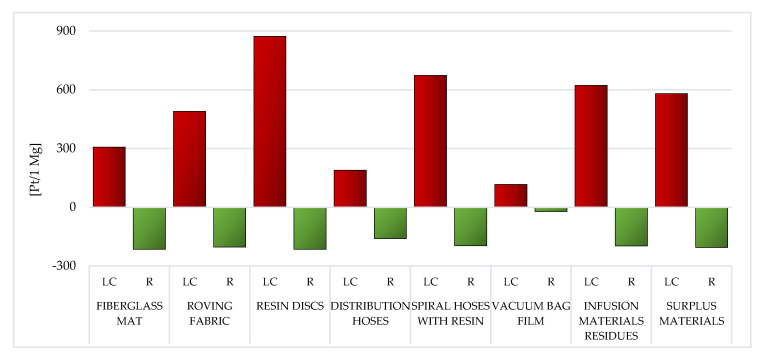
Grouping and weighting outcome of milieu repercussions appearing in the LC of chosen post-production waste of WPPB: LC—life cycle, R—recycling [Pt/1 Mg].

During the analysis of individual types of WPPB post-production waste, it is visible that the elevated entire volume of potential negative influence on the environment is characteristic of the resin discs LC (total: 873.04 Pt/1 Mg). The production of epoxy resins involves an enormous demand for energy and raw materials, and thus the high result recorded for this type of waste. This also translates into a high volume of potential influence on the milieu of spiral hoses with resin, which, although made of PVC, nevertheless, after the vacuum infusion processes are completed, epoxy resin remains in them, which solidifies (total: 673.78 Pt/1 Mg) (Figure 6).

The employ of recycling processes could lessen the volume of detrimental impacts over the LC of a given type of WPPB usually by approximately one-third—from ~18% for vacuum bag film (made of co-extrusion of polyolefin and nylon based resins), to ~85% for distribution hoses (made of PVC and polyester fiber) (Figure 6).

So as to achieve better identification of the areas of the LC of individual post-production WPPB, which may hold the substantial obstructive (or constructive) influence on the milieu, a thorough evaluation of substances and processes arising inside the eleven considered impact categories was executed.

##### Carcinogens

**Figure 7:**
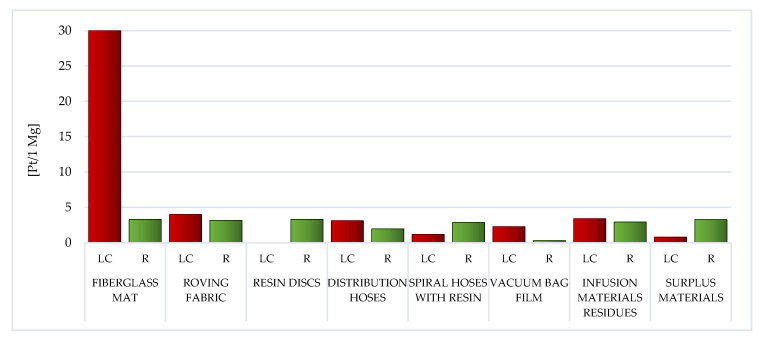
Grouping and weighting ramifications of milieu effects for carcinogenic compounds available in designated post-production waste of WPPB: LC—life cycle, R—recycling [Pt/1 Mg].

The elevated volume of prospective detrimental outcome on human health of carcinogenic compounds was recorded in the LC of fiberglass mat (total: 30.03 Pt/1 Mg), made of chopped glass fiber. During the production of this material, in particular cadmium (15.69 Pt/1 Mg) and arsenic (8.56 Pt/1 Mg) are emitted to the atmosphere. Arsenic can cause liver and bronchial cancer. In turn, cadmium is a toxic element that damages the kidneys, causes bone and cardiovascular diseases and may interfere with the development of fetuses (Figure 7; Table S1) [59].

The lowest effect of carcinogenic compounds was characteristic of resin discs made of epoxy resin (gross: 0.03 Pt/1 Mg). Basically, these were the emissions of some metallic ions (0.02 Pt/1 Mg) due to resin production (Figure 7; Table S1 in Supplementary Information).

Recycling of waste originated through the manufacture of WPPB is affiliated with small emissions of carcinogenic compounds (involving those generated through chemical processes occurring as a conclusion of the employ of reagents used in such processes). The lowest volume of influence in this area was documented for the recycling of vacuum bag film (0.33 Pt/1 Mg), while the highest for fiberglass mat (3.31 Pt/1 Mg). However, the employ of recycling processes to some range permits to diminish the emissions of chosen carcinogenic compounds in the perspective of the entire LC of the analyzed waste, in particular, some metallic ions (from −0.18 Pt/1 Mg for fiberglass mat, resin discs and surplus materials, to −0.02 Pt/1 Mg for vacuum bag film) (Tables S1 and S2).

##### Organic Compounds Causing Respiratory Diseases

**Figure 8:**
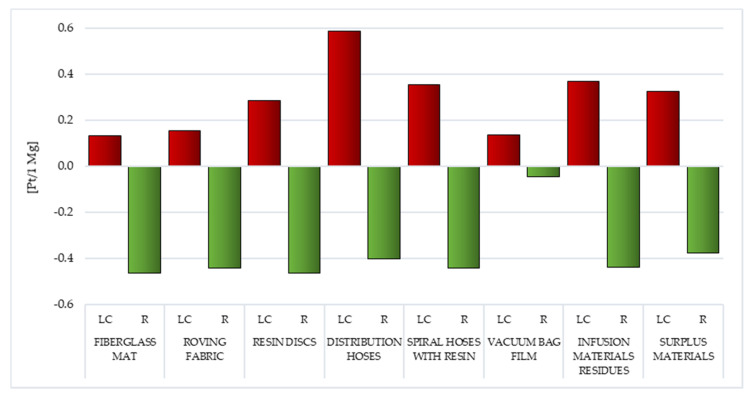
Grouping and weighting ramifications of milieu effects for organic compounds causing respiratory diseases available in designated production waste of WPPB: LC—life cycle, R—recycling [Pt/1 Mg].

Among the considered variety of waste induced during the manufacture of WPPB, the LC of distribution hoses made of PVC and polyester fiber (gross 0.59 Pt/1 Mg) is characterized by the elevated volume of potential emissions of organic compounds causing respiratory system diseases. They are mainly the emissions of some hydrocarbons related to PVC production processes. Hydrocarbons are the key constituent of crude oil. Their emissions to the atmosphere can cause a number of respiratory diseases, including increasing the incidence of asthma (Figure 8; Table S3) [59].

Compared to the remaining analyzed waste, the LC of fiberglass mat and vacuum bag film are distinguished by the lowest volume of probable hazardous impacts on human health in the considered impact category, amounting to a total of 0.13 Pt/1 Mg (Figure 8; Table S4).

In the case of waste with a predominant material composition of fiberglass (like fiberglass mat and roving fabric), the highest negative impact was recorded for non-methane volatile organic compounds (in sequence 0.1 and 0.14 Pt/1 Mg), which are generated during the production of fiberglass. For the remaining waste considered, the main component of emissions of organic substances causing respiratory diseases are the previously mentioned hydrocarbons—from 0.07 Pt/1 Mg for vacuum bag film, to 0.57 Pt/1 Mg for distribution hoses (Tables S3 and S4).

Reuse of WPPB waste, for example, in the production of other products, can become a potential source of environmental and health benefits, mainly in the area of reducing emissions to the atmosphere non-methane volatile organic compounds (from −0.05 Pt/1 Mg for vacuum bag film, to −0.47 Pt/1 Mg for fiberglass mat and resin discs). Recycling fiberglass mat and resin discs allows to reduce the volume of the probable influence on human health of the emission of organic compounds causing respiratory diseases by 0.46 Pt/1 Mg, in terms of their entire LC (Figure 8; Tables S3 and S4).

##### Inorganic Compounds Causing Respiratory Diseases

**Figure 9:**
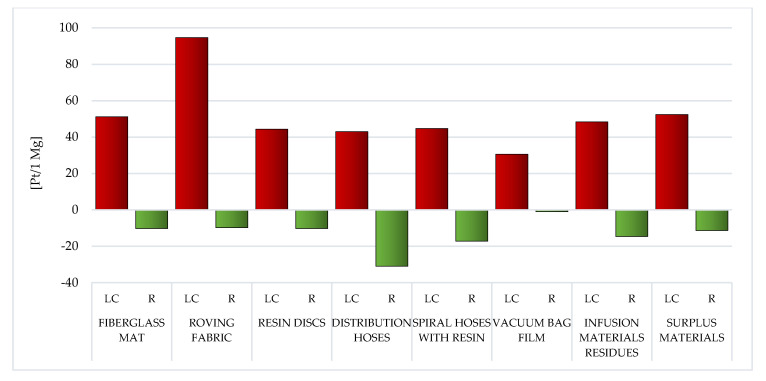
Grouping and weighting ramifications of milieu effects for inorganic compounds causing respiratory diseases available in designated production waste of WPPB. LC: life cycle, R: recycling [Pt/1 Mg].

As mentioned before, one of the two impact categories distinguished by the elevated volume of prospective detrimental outcomes on the environment is the emission of inorganic compounds causing respiratory diseases. In this area, roving fabric (total: 94.75 Pt/1 Mg) made of epoxy silane-coated fiberglass can cause the most damage to human health. The use of epoxy silanes, on the one hand, improves the properties of composites, but on the other hand, the processes of their production are characterized by a higher level of negative environmental impact (Figure 9 and Table S5).

The LC of a vacuum bag film made of co-extrusion of polyolefin and nylon-based resins is distinguished by the lowest volume of prospective detrimental outcomes on living organisms in the area under consideration (total: 30.56 Pt/1 Mg). However, in the case of this material, the use of recycling processes is associated with a reduction of harmful impacts in the viewpoint of the undivided LC by only about 3.5%, which is the lowest percentage compared to other types of post-production waste WPPB (Tables S5 and S6).

In the field of inorganic compounds causing respiratory diseases, in the case of almost all analyzed types of waste, the greatest share in the total value of the potential harmful effect on the health was due to emissions to the atmosphere of nitrogen oxides (from 18.48 Pt/1 Mg for fiberglass mat, to 39.19 Pt/1 Mg for resin discs) and sulfur dioxide (from 1.36 Pt/1 Mg for spiral hoses with resin, to 28.15 Pt/1 Mg for roving fabric). Nitrogen oxides is one of the most dangerous substances emitted into the atmosphere, incl. during the production processes of polymer composites. It is considered to be almost ten times more harmful to health than carbon monoxide, and several times more harmful than sulfur dioxide. It is a cause of chronic bronchitis and emphysema; it also increases the susceptibility to respiratory infections. SO_2_ is a by-product of the combustion of fossil fuels; it strongly irritates the respiratory system, is toxic to humans and animals, and is harmful to plant growth and development (Tables S5 and S6) [59].

The use of recycling processes in relation to the considered WPPB waste can meaningfully lessen the prospective detrimental influence on the milieu in the perspective of their undivided LC. The highest level of lessening was recorded in the case of nitrogen oxides (from −1.39 Pt/1 Mg for vacuum bag film, to −23.25 Pt/1 Mg for distribution hoses) (Tables S5 and S6).

##### Climate Change

**Figure 10:**
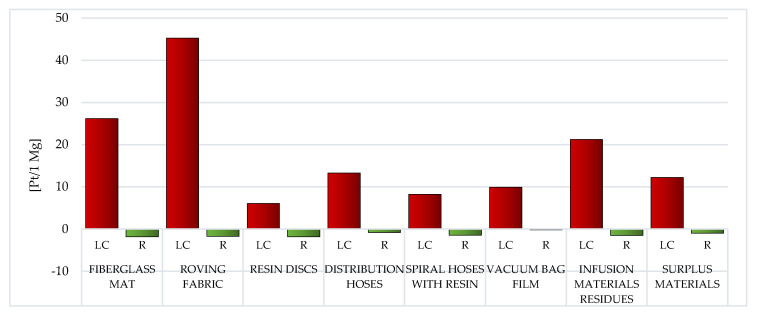
Grouping and weighting ramifications of milieu effects for compounds causing climate change available in designated production waste of WPPB: LC—life cycle, R—recycling [Pt/1 Mg].

Today, climate change is the most urgent environmental problem worldwide. It is most often considered in the perspective of global warming caused by greenhouse gas (GHG) emissions. The maximum potential negative impact on the milieu of compounds causing climate change was documented within the LC of roving fabric (gross: 45.26 Pt/1 Mg) and fiberglass mat (gross: 26.19 Pt/1 Mg), with the largest mass share of fiberglass. The procedure accompanying the manufacture of fiberglass require a lot of energy, which usually comes from the combustion of conventional fuels. Consequently, it is associated not only with the depletion of non-renewable resources, but also with a demeaning of the environmental quality. This in turn directly contributes to the emergence of many different health problems in humans and animals. The lowest impact on climate change is in the LC of resin discs (total: 6.05 Pt/1 Mg) made of epoxy resin (Figure 10; Table S7).

CO_2_ is the key compound shaping the size of the potential adverse impact on climate change in the LC of all the considered WPPB waste. It has a crucial role in the greenhouse effect. Its concentration varies seasonally and depending on latitude (changes can also be noted locally, especially near the Earth’s surface). The employ of recycling as a format of post-use management of waste generated during the manufacture of WPPB allows to reduce the harmful effects of compounds causing climate change, in particular CO_2_, in the perspective of the entire LC (Tables S7 and S8) [60].

##### Radiation

During the manufacture of WPPB and the related operations of obtaining fossil raw materials, significant quantities of materials and energy are used. As already mentioned, energy is most commonly obtained from conventional energy sources, mainly inter alia from coal. During the combustion of coal, dust and gases are emitted to the atmosphere, containing not only harmful substances, such as sulfur oxides, nitrogen oxides, mercury vapors, chlorine, fluorine and heavy metals, but also trace elements that have natural radioactivity. These elements include uranium, thorium and the countless products of their decomposition, including radium and radon. While these elements are chemically reduced to less harmful forms than some different components (for instance, arsenic, selenium or mercury), there is a problem with some radiation-related health risks [61]. 

**Figure 11:**
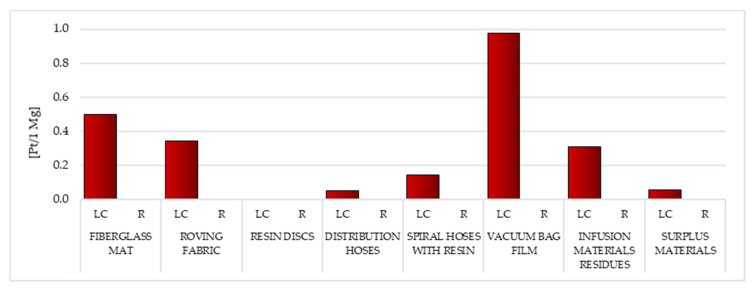
Grouping and weighting ramifications of milieu effects for radioactive substances available in designated production waste of WPPB: LC—life cycle, R—recycling [Pt/1 Mg].

The post-production waste with the elevated prospective volume of detrimental influence of radioactive substances on the milieu is vacuum bag film (total: 0.97 Pt/1 Mg), and the lowest is distribution hoses and surplus materials (total: 0.05 Pt/1 Mg). To the greatest extent, the level of this type of influence consists of emissions of the isotope ^222^ Radon, which occurs in the LC of almost entire evaluated WPPB (from 0.03 Pt/1 Mg for distribution hoses, to 0.82 Pt/1 Mg for vacuum bag film) (Figure 11; Tables S9 and S10).

##### Ozone Layer

**Figure 12:**
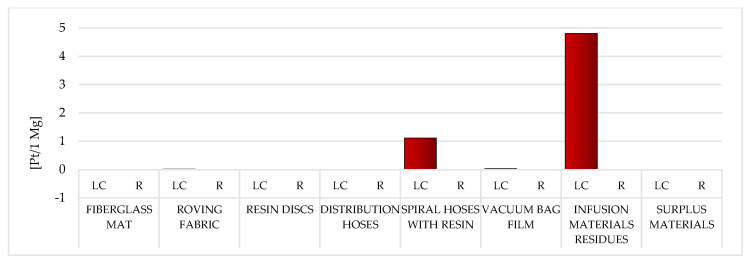
Grouping and weighting ramifications of milieu effects for compounds causing an ozone hole enlargement available in designated production waste of WPPB: LC—life cycle, R—recycling [Pt/1 Mg].

Analyzing the aspects related to reducing environment quality and, consequently, its influence on human health, the problem of the ozone hole enlargement is also important. Stratospheric ozone absorbs some of the ultraviolet radiation that reaches the Earth. Certain types of radiation are harmful to living organisms because they can damage cells and the genetic material they contain. In humans and animals, for example, neoplastic changes may appear. Therefore, it is reasonable to use measures to prevent the ozone (O_3_) concentration drop in the stratosphere. Comparing the LC of the analyzed WPPB, it can be noticed that infusion material residues and spiral hoses with resin are distinguished by the elevated level of potential emissions of compounds causing the ozone hole enlargement (in sequence: 4.81 and 1.12 Pt/1 Mg). The remaining waste does not have a significant impact in this area (Figure 12; Tables S11 and S12) [62].

The amount of this harmful effect consists mainly of emissions of tetrachloromethane (CFC-10), which is sometimes used as a solvent in organic chemistry and a refrigerant. Long-term exposure to CFC-10 may add to the development of diseases of the central nervous system, liver and kidneys, and as a consequence even lead to death (Tables S11 and S12) [50].

##### Ecotoxicity

**Figure 13:**
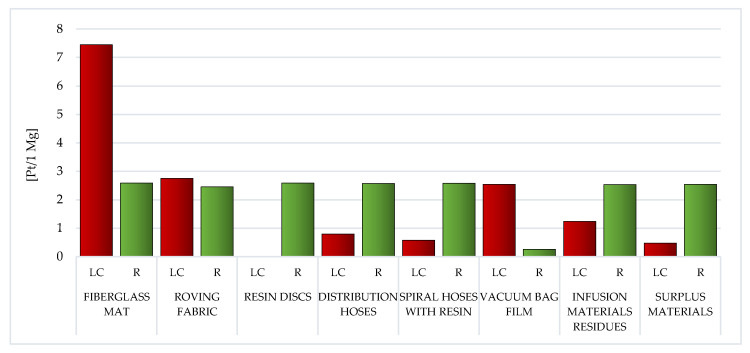
Grouping and weighting ramifications of milieu effects for ecotoxic compounds available in designated post-production waste of WPPB: LC—life cycle, R—recycling [Pt/1 Mg].

Among the analyzed variety of waste induced through the manufacture of WPPB, the LC of fiberglass mat made of chopped glass fiber (gross: 7.45 Pt/1 Mg) is distinguished by the elevated volume of potential emissions of ecotoxic compounds. It consists mainly of cadmium and nickel emissions related to production processes (in sequence 3.36 and 2.24 Pt/1 Mg). Fossil fuels (like hard coal) contain significant amounts of cadmium. As a result of their extraction and conversion, it is emitted to the atmosphere, hydrosphere and soil (Figure 13; Table S13) [61].

Compared to other analyzed waste, the LC of resin discs made of epoxy resin is distinguished by the lowest volume of potential hazardous effects on human health in the considered impact category, not exceeding 0.01 Pt/1 Mg (Figure 13 and Table S13).

For most of the analyzed wastes, the highest negative impact was noted for nickel (from 2.24 for fiberglass mat to <0.01 Pt/1 Mg for resin discs). Nickel is used as a catalyst for many reactions, including its usage in the production of polymers. Excess nickel in the human body has a toxic and carcinogenic effect. Excessive inhalation of airborne nickel compounds can cause bronchial asthma and pneumoconiosis, and can also damage the kidneys, liver, spleen and brain (Tables S13 and S14) [59].

Recycling of waste induce through the manufacture of WPPB is analogous with low emissions of ecotoxic compounds (containing these generated during chemical reactions occurring as a consequence of the employ of reagents used in such processes). The lowest volume of influence in this area was documented for the vacuum bag film recycling processes (0.26 Pt/1 Mg), while for other waste it was comparable and was within from 2.46 Pt/1 Mg for roving fabric to 2.59 Pt/1 Mg for fiberglass mat (Tables S13 and S14).

##### Acidification/Eutrophication

**Figure 14:**
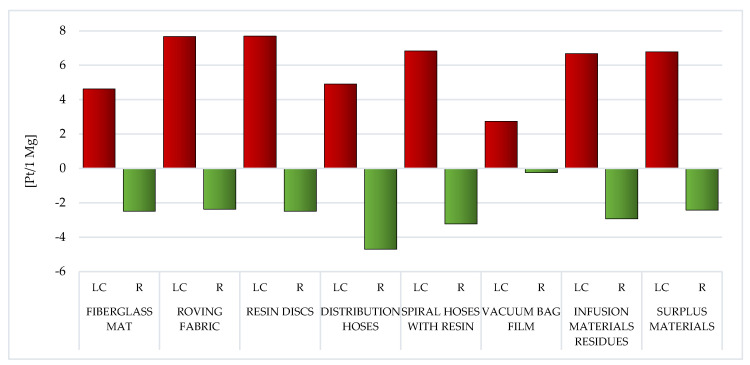
Grouping and weighting ramifications of milieu consequences for acidifying or eutrophication compounds available in designated production waste of WPPB: LC—life cycle, R—recycling [Pt/1 Mg].

The compounds that possess a remarkable influence on the reduction of the environment quality includes, among others those that cause acidification or eutrophication. They occur in the LC of all WPPB, but resin discs (total: 7.7 Pt/1 Mg) and roving fabric (gross: 7.67 Pt/1 Mg) may pose a particular danger in this regard. Subsequently, the lowest level of harmful effects within this impact category was documented for vacuum bag film (total: 2.74 Pt/1 Mg) (Figure 14 and Table S15).

Post-use management of the analyzed waste can potentially lessen the detrimental influence on the milieu of compounds causing acidification or eutrophication by about 10% (for vacuum bag film), even up to about 95% (for distribution hoses), in the standpoint of their whole LC (Tables S15 and S16).

Both the highest level of potential harmful effects throughout the LC and the elevated level of probable easing due to the use of recycling processes are noticeable for the same substance: nitrogen oxide. Its emission in the LC of all the considered post-production waste of WPPB causes a row influence from 7.56 Pt/1 Mg for resin discs to 1.86 for vacuum bag film. Recycling can reduce its potential negative effects by from −4.48 Pt/1 Mg for distribution hoses to −0.27 Pt/1 Mg for vacuum bag film. Nitrogen oxide is a harmful component of exhaust gases and many industrial pollutants. It is one of the most dangerous compounds polluting the atmosphere, it reacts easily and it is the main culprit that causes smog. It is also associated with the formation of the greenhouse effect and the phenomenon of acid rain acidifying the soil and water. It influences the development of many crucial diseases, for instance, bronchial asthma, chronic obstructive pulmonary disease, diseases of the cardiovascular system and cancer (in particular of the lungs), thus the great importance of actions aimed at reducing the emission of this chemical compound to the environment (Tables S15 and S16) [59].

##### Land Use

**Figure 15:**
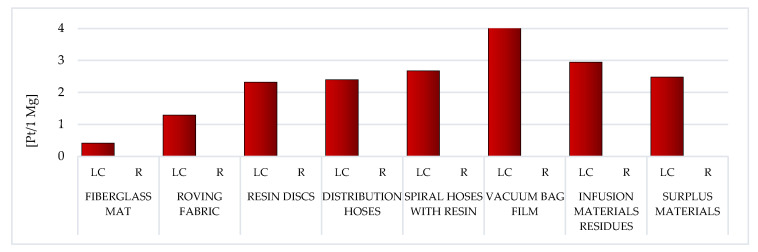
Grouping and weighting ramifications of milieu effects for land use processes available in designated post-production waste of WPPB: LC—life cycle, R—recycling [Pt/1 Mg].

The individual phases of the LC of the appraised waste, resulting from the manufacture of WPPB, are inextricably linked with the necessity to land use, both in the factors of the extraction of fossil fuels and mineral resources, and their subsequent processing. The highest level of potential harmful impact below this impact category is visible for vacuum bag film and amounts to a total of 4.02 Pt/1 Mg. This value is mainly influenced by land use (class II-III)—3.57 Pt/1 Mg. The lowest potential negative impact in this area was recorded for fiberglass mats (total: 0.42 Pt/1 Mg) (Figure 15; Tables S17 and S18).

For most of the analyzed waste types, the most important harmful process related to land use is the occupation of an industrial area (from 0.02 Pt/1 Mg for roving fabric to 2.36 Pt/1 Mg for distribution hoses). The creation of an industrial infrastructure is an indispensable element of any production activity, and it is associated with the sustainable use of land, leading to hardly reversible changes in the soil environment and usually also in the water environment (mainly groundwater) (Tables S17 and S18).

##### Minerals

Each production process, including WPPB, involves the consumption of a certain amount of energy and matter. In order to produce materials and components, it is necessary to first extract the appropriate raw materials, which also involves a certain need for energy and auxiliary substances. One of the key raw materials for the production of polymers is, among other organic materials and natural resources, such as cellulose, coal, natural gas and, of course, crude oil. Nevertheless, in the procedure of their production, it also becomes mandatory to bestow a definite, usually small, portion of mineral resources.

**Figure 16:**
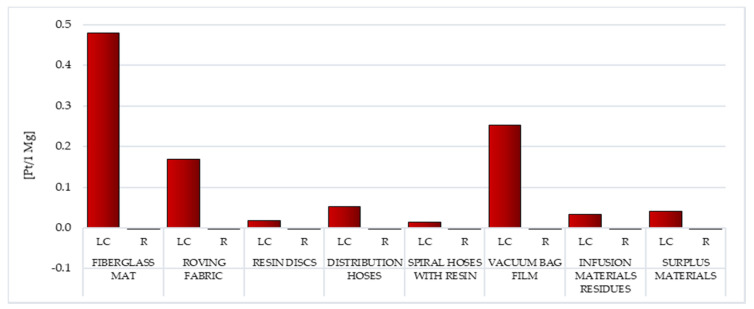
Grouping and weighting ramifications of milieu effects for operations affiliated to the extraction of mineral resources available in designated post-production waste of WPPB: LC—life cycle, R—recycling [Pt/1 Mg].

The highest volume of probable hazardous environmental impact of undertaking affiliated to the extraction of mineral resources was documented for the LC of fiberglass mat (gross: 0.48 Pt/1 Mg), vacuum bag film (total: 0.25 Pt/1 Mg) and roving fabric (gross: 0.17 Pt/1 Mg). Therefore, is a visible increase in the degree of influence below this impact category for waste made primarily of fiberglass and nylon (Figure 16; Tables S19 and S20).

The most negative environmental influence can be precipitated by procedure affiliated to the acquisition of aluminum (up to a maximum of 0.46 Pt/1 Mg for fiberglass mat) and copper (up to an utmost of 0.22 Pt/1 Mg for vacuum bag film). The aluminum production process initiates with the assembly of an opencast mine to mine bauxites. Copper is also most often mined this way. Opencast mining causes a downgrading of the environment quality, including through transformations of the terrain surface, destruction of the soil cover in the mining area, changes in water conditions, air pollution, excessive noise emission, seismic shocks, impact on the animated nature (flora and fauna), the generation of waste and sewage from the mine’s activities and drainage (Tables S19 and S20) [61].

##### Fossil Fuels

**Figure 17:**
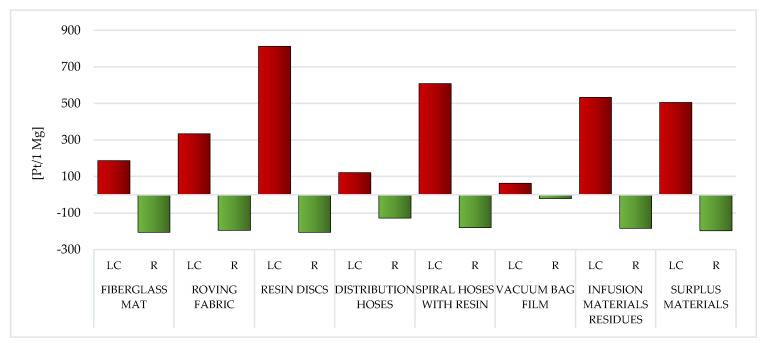
Grouping and weighting ramifications of milieu effects for the operations affiliated to the extraction of fossil fuels available in designated post-production waste of WPPB: LC—life cycle, R—recycling [Pt/1 Mg].

As previously disclosed, the main fossil fuels used in the manufacture of polymer materials are crude oil, coal and natural gas. The procedure affiliated to their extraction outstandingly diminishes environment quality. The elevated volume of probable obstructive influence in this impact category was documented for the LC of resin discs made of epoxy resin (total: 812.31 Pt/1 Mg), while the lowest for vacuum bag film made of co-extrusion of polyolefin and nylon based resins (gross: 62.69 Pt/1 Mg) (Figure 17, Tables S21 and S22).

The level of the total harmful influence of the LC of individual WPPB was primarily influenced by the processes related to the extraction of crude oil (up to a maximum of 736.71 Pt/1 Mg for resin discs) and natural gas (up to a maximum of 209.73 Pt/1 Mg for roving fabric). Increasingly faster consumption of crude oil, natural gas and coal directs moreover to the draining of these non-renewable energy sources—their exploitation also poses abundant problems related to milieu degeneration. The fuel crisis and technological progress have started the era of oil sands exploitation in the form of opencast mines. The techniques for obtaining these raw materials, due to the properties of the deposit and their location, are more harmful to the environment than the traditional extraction of natural gas or liquid crude oil and causes practically irreversible damage. It is assumed that in an opencast mine, obtaining each barrel of oil usually requires first cutting the forest, then removing about 2 tons of peat and soil that covers the oil sands, and finally extracting 2 tons of sand alone. Extraction of tar sand and obtaining a barrel of oil from it results in approximately three times more CO₂ emissions to the atmosphere than extraction of a barrel from traditional deposits, like, for example, in Saudi Arabia. In addition, the water used in the oil extraction process is a post-production waste that is located near the mine (in the form of a reservoir that is dangerous to the environment and health). Post-production wastewater from open pit oil sands contains carcinogenic substances like cadmium, lead, sulfur, zinc, naphthenic acid or polycyclic aromatic hydrocarbons, which may contaminate groundwater and, in result, affect human health (Tables S21 and S22) [61,63].

Recycling of WPPB post-production waste allows not only to reduce the decrease of non-renewable resources, but also to outstandingly devalue the natural environment. The recycling processes of fiberglass mats make it possible to reduce the volume of probable detrimental repercussions of technique correlated to the extraction of fossil fuels to the greatest extent, in the angle of the total LC of the examined waste (gross: −204.85 Pt/1 Mg). The magnitude of the reduction of negative impacts varies depending on the materials of the waste. Recycling can result in not only the complete elimination of harmful effects in the perspective of the waste LC, but even in an increase in the level of environmental quality, as it can be the case with fiberglass mat and distribution hoses (thanks to energy and matter savings compared to production from primary raw materials) (Tables S21 and S22).

The most problematic waste in terms of the scrutinized impact category is resin discs. Their LC generates the elevated volume of potential negative influence on the environment, and recycling processes to a very small extent, can reduce this harmful impact (only ~25%) (Table S21).

#### 3.1.2. Areas of Influence

As mentioned before, the modeling ramifications using the Eco-Indicator 99 were split up into two groups. Section 3.1.1 presents the results obtained for eleven impact categories, while Section 3.1.2 presents the results covering the three areas of influence (human health, ecosystem quality and resources). Particular attention was furthermore focused to the areas of emission of sole chemical compounds (air, water, soil and raw). Similarly to Section 3.1.1, the results are summarized separately for the LC and for the recycling processes of eight analyzed wastes from the production of WPPB.

##### Human Health

**Figure 18:**
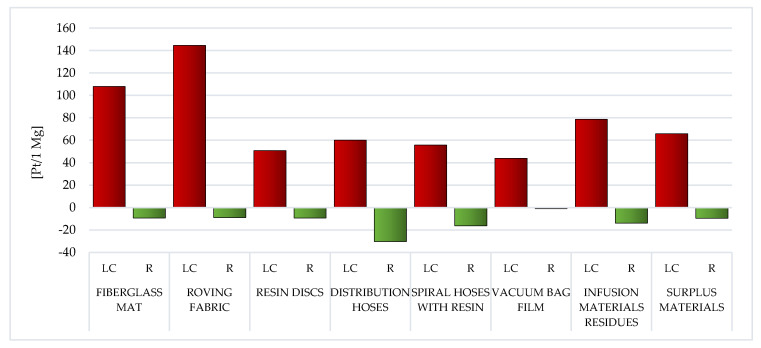
Grouping and weighting ramifications of milieu effects for substances harmful to human health, available in designated post-production waste of WPPB: LC—life cycle, R—recycling [Pt/1 Mg].

Among the assessed post-production waste of WPPB, the roving fabric (total: 144.53 Pt/1 Mg) and fiberglass mat (gross: 108.02 Pt/1 Mg) LC have the highest probable negative influence on human health. A significantly higher degree of harmful effects of waste, which are mainly produced on the groundwork of fiberglass, is visible. The LC of a vacuum bag film is characterized by the lowest amount of emissions of substances that may adversely affect human and animal health (total: 43.92 Pt/1 Mg) (Figure 18, Tables S23 and S24).

In the case of most of the analyzed post-production waste of WPPB, the factor that most affects the size of the negative impact of their LC on health, is nitrogen oxides emission (from 9.65 Pt/1 Mg for vacuum bag film to 39.19 Pt/1 Mg for resin disc). The deviation is roving fabric, where such factor is carbon dioxide (40.19 Pt/1 Mg) and vacuum bag film—where it is SO_x_ (15.27 Pt/1 Mg). One of the key milieu issues affiliated to the pursuit of the economic sector including polymeric materials is emissions of toxic substances (and in some cases also wastewater with potentially high loads of organic compounds), relatively large amounts of solvents used, significant waste production, as well as enormous energy demand. Industrially, nitrogen oxides are most often obtained by the oxidation of ammonia. It is used in the production processes of many polymers. However, it has an obstructive influence on human health, irritating the respiratory system and can cause a number of diseases in this area (like chronic bronchitis or emphysema) (Tables S23 and S24) [59].

The application of recycling procedures for wholly considered wastes may cause (to a greater or lesser extent) a reduction in the level of negative impacts on human health in the perspective of their total LC. The highest volume of downgrading was characteristic for distribution hoses (gross: −30.21 Pt/1 Mg). This value has the greatest impact on the reduction of nitrogen oxides emissions in the instance of each of the evaluated waste of WPPB. (from −1.39 Pt/1 Mg for vacuum bag film to −23.25 Pt/1 Mg for distribution hoses) (Tables S23 and S24).

**Figure 19:**
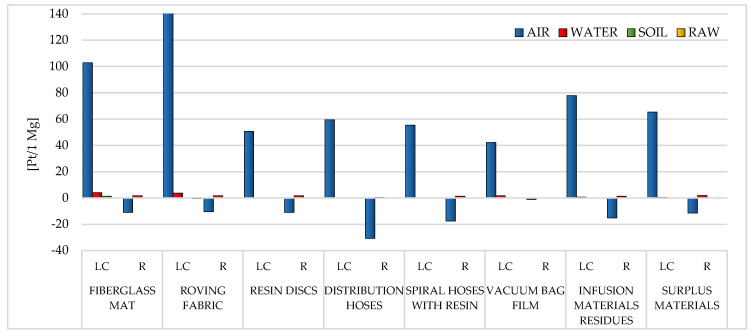
Grouping and weighting ramifications of milieu effects for the areas of emission of substances harmful to human health, available in designated post-production waste of WPPB: LC—life cycle, R—recycling [Pt/1 Mg].

The greatest amount of matter of high risk to human health is emitted to the atmospheric environment. The roving fabric LC characterizes a particular risk in this aspect (total emissions to air: 141.23 Pt/1 Mg) and the fiberglass mat (gross emissions to air: 102.67 Pt/1 Mg). The elevated volume of emissions to the aquatic environment was also recorded for the LC of the fiberglass mat (total emissions to water: 4.16 Pt/1 Mg) and the roving fabric (gross emissions to water: 3.79 Pt/1 Mg). However, a significant level of emissions to soil occurs in the case of the fiberglass mat LC (total emissions to soil: 1.36 Pt/1 Mg) and the distribution hoses (total emissions to soil: 0.14 Pt/1 Mg). Due to the use of recycling processes, the greatest benefits for human health, in the perspective of the total LC of WPPB, were recorded in one compartment: air. The maximum level of potential reduction could be for the distribution hoses (total reduction of air emissions: −30.73 Pt/1 Mg) and the spiral hoses with resin (gross reduction of air emissions: −17.56 Pt/1 Mg) (Figure 19).

##### Ecosystem Quality

**Figure 20:**
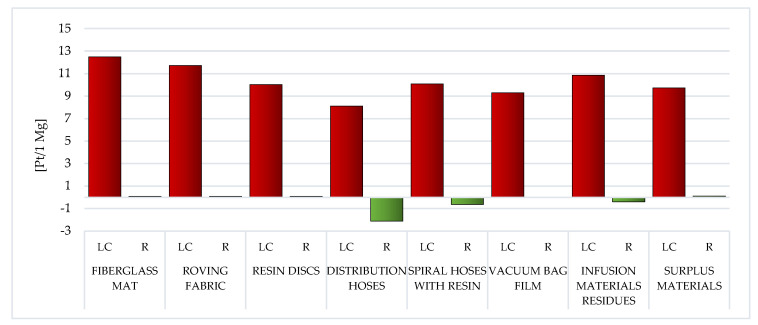
Grouping and weighting ramifications of milieu effects for substances and procedures lowering the quality of the environment available in designated post-production waste of WPPB: LC—life cycle, R—recycling [Pt/1 Mg].

In addition to substances determining human health, in the LC of waste fabricated during the manufacture of WPPB, there are also processes and chemical compounds that narrow of the environment quality. The negative impact on the environment will also significantly affect the deterioration of human and animal health. Among all the considered wastes, the substantial probable detrimental influence on the quality of the milieu has those that contain the highest share of fiberglass, that is, the fiberglass mat (total: 12.49 Pt/1 Mg) and the roving fabric (total: 11.72 Pt/1 Mg). Afterwards, the smallest negative impact in this area of influence is characteristic of the distribution hoses existence cycle (total: 8.11 Pt/1 Mg) (Figure 20, Tables S25 and S26).

The level of nitrogen oxides emission, which is of key importance for the above-mentioned values, is from 7.56 Pt/1 Mg for resin discs to 3.56 Pt/1 Mg for the fiberglass mat. Nitrogen oxide is one of the causes of smog, mainly photochemical; it causes soil acidification, which in turn intensifies the production of toxic compounds (including carcinogens) that penetrate into plants. In the case of fiberglass mat, cadmium emissions to the atmosphere are the second most important factor (3.36 Pt/1 Mg). Cadmium is associated with modern fiberglass production processes, but due to its extreme toxicity, it is not only a health hazard, but also reduces the quality of the environment. The presence of cadmium in the environment of humans and animals, the ability to accumulate in the body, long biological half-life (estimated at 15–40 years in humans) and direct or indirect toxic effects, resulting in cell damage and disturbance of their vital functions, posing a serious threat. In the analyzed area of influence, the only exception is the vacuum bag film, which generates the most negative environmental consequences due to land use associated to the extraction of mineral resources, fossil fuels and production processes (including the necessary technical infrastructure) (Tables S25 and S26) [62].

Throughout the LC, recycling processes can improve the quality of the environment. The highest level of potential reduction of the harmful impact on the environment, in relation to all analyzed wastes, stands out for the distribution hoses (total: −2.12 Pt/1 Mg). The amount of this reduction (for almost all considered wastes) is primarily influenced by the reduction of the negative impact of the nitrogen oxides emissions (from −2.5 Pt/1 Mg for surplus materials to −4.48 Pt/1 Mg for distribution hoses). The sole deviation is the vacuum bag film, for which the greatest reduction in harmful influences was noted for the nitric oxide (−0.27 Pt/1 Mg) (Tables S25 and S26).

**Figure 21:**
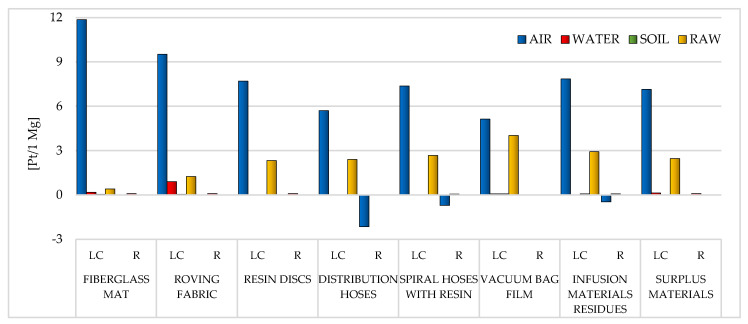
Grouping and weighting ramifications of milieu effects for the areas of emission of substances and processes lowering the quality of the environment, present in selected post-production waste of WPPB: LC—life cycle, R—recycling [Pt/1 Mg].

The emissions to the atmosphere have the greatest influence on the milieu of the LC of WPPB. Their level is particularly high in the case of waste mainly made of fiberglass, that is the fiberglass mat (total emissions to air: 11.86 Pt/1 Mg) and the roving fabric (gross emissions to air: 9.52 Pt/1 Mg). Another important group are waste materials, the maximum amount of which accompanies the LC of the vacuum bag film (gross raw emissions: 4.01 Pt/1 Mg). In the case of emissions to water, the most harmful emissions were recorded for the roving fabric (total emissions to water: 0.9 Pt/1 Mg), and regarding emissions to soil, for the vacuum bag film (total emissions to soil: 0.08 Pt/1 Mg). Recycling techniques can diminish the negative environmental influence of the analyzed waste LC, especially in the area of emissions to the atmosphere (maximum reduction of air emissions: −2.14 Pt/1 Mg for distribution hoses) (Figure 21).

##### Resources

**Figure 22:**
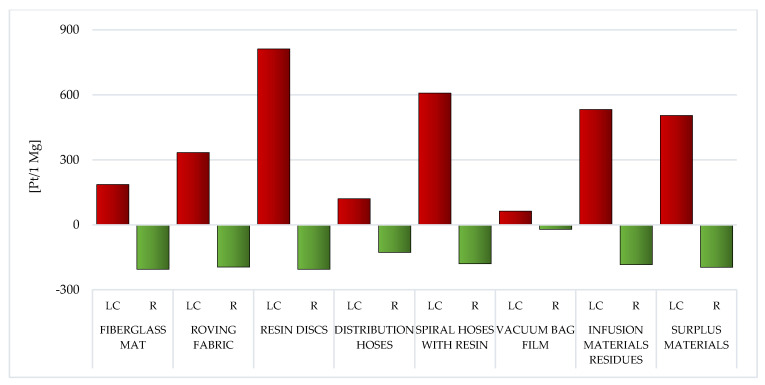
Grouping and weighting ramifications of milieu effects for procedures associated to the depletion of raw materials, available in designated post-production waste of WPPB: LC—life cycle, R—recycling [Pt/1 Mg].

Of all the areas of influence, two resources have the largest share in the overall environmental impact of WPPB. Both mineral raw materials and fossil fuels form the basis of the production processes of all polymers. The elevated volume of potential detrimental effect in this area was documented for the LC of the resin discs (total: 812.33 Pt/1 Mg) and the spiral hoses with resin (gross: 608 Pt/1 Mg). The common factor for both of these wastes is the high epoxy resin content. The LC is characterized by the lowest volume of obstructive influence of the vacuum bag film made of co-extrusion of polyolefin and the nylon-based resins (gross: 62.94 Pt/1 Mg) (Figure 22, Tables S27 and S28).

In the case of waste, the composition of which is mainly based on fiberglass, the extraction of the natural gas (~110 Pt/1 Mg for fiberglass mat and ~210 Pt/1 Mg for roving fabric). On the other hand, in relation to other waste, the processes of the crude oil extraction (from 736.72 Pt/1 Mg for resin discs to 46.61 for vacuum bag film) are significantly more important. As already mentioned in Fossil Fuels, in Section 3.1.1, these are processes mainly based on surface mining, causing enormous damage to the natural environment (Tables S27 and S28).

Recycling of WPPB post-production waste would significantly lessen the volume of obstructive influence of their LC in the area of resource depletion (from ~110% for the fiberglass mat to ~30% for the spiral hoses with resin). The highest unit reduction level, amounting to −204.86 Pt/1 Mg, was documented for the fiberglass mat and the resin discs, while the lowest, equal to −20.49 Pt/1 Mg for the vacuum bag film. Especially good effects in this area of influence can be obtained in the field of processes related to the extraction of crude oil and natural gas. The recycling of polymers has become an extremely important issue in the modern economy, not only due to the aspects directly related to environmental protection, but also the growing problem of depletion of nonrenewable resources. The demand for various types of polymers increases every year, thereof is an urgent need for an effective solution to this problem (Tables S27 and S28).

### 3.2. CED

As mentioned before, the outcome of the scrutiny implemented as fragment of the LCIA are summarized in two sections: the 1st one covered the Eco-Indicator 99 method (Section 3.1), followed by the CED method. All the results obtained in the CED analyzes were also presented in the Pt per 1 Mg unit. Similar to the previous sections, the obtained results are presented separately for the LC and for the recycling technique of 8 considered wastes from the WPPB production procedure.

**Figure 23:**
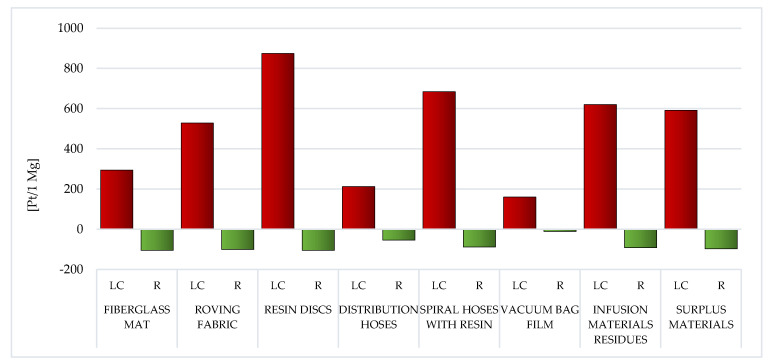
Grouping and weighting results of environmental consequences for energy production processes for appointed post-production waste of WPPB: LC—life cycle, R—recycling [Pt/1 Mg].

The resin disc LC incorporates numerous energy-demanding actions (especially at the stage of raw material extraction and production), compared to all other analyzed post-production waste. As they are usually correlated with the application of energy attained from non-renewable sources, the volume of potential detrimental environmental impact of processes related to energy production is the highest for the LC of this waste and amounts to a total of 874.33 Pt/1 Mg. The nether volume of energy demand is found in the LC of a vacuum bag film, which translates into a potential impact of 160.93 Pt/1 Mg (Figure 23; Table 3 and Table 4).

Recycling processes would allow to limited expanse to diminish the harmful influence of processes related to energy production in the stand of the entire LC of the analyzed waste, principally for the fiberglass mat and the resin discs (gross: −105.23 Pt/1 Mg). The highest volume of obstructive influence reduction would be for non-renewable energy sources. Unfortunately, the portion of renewable energy in the LC of plastics, materials and WPP is still very low (Table 3 and Table 4).

## 4. Discussion

Control of waste and other negative consequences of the manufacture of WPPB is possible and becomes a necessity for sustainable development through the use of polymer materials. The world economy is based on fossil fuels. Although they are a very efficient source of energy, easy to store and use at any time, using them also has many negative consequences. Environmental damage related to their extraction, emissions of hazardous substances, climate change, extinction of species, ecological disasters or geopolitical problems related to conflicts over resources are among the many threats posed by the exploitation of non-renewable resources. A global economy, exponentially growing, based on an infrastructure dependent on fossil fuels has been created, and global GDP, energy consumption and CO_2_ emissions are increasing at a similar rate [63,64,65].

The easy-to-extract oil, coal and gas reserves have been largely exhausted. In order to maintain and power the current system, more and more hard-to-reach deposits are being used—from Arctic oil to oil sands and shale gas. Therefore, it becomes necessary to search for alternative energy sources. One of the sectors of renewable energy is wind energy. During the functioning of WPP, almost no harmful chemical compounds are emitted into the milieu. Nonetheless, their manufacture and post-use management after end-of-life are associated with many environmental hazards that are rarely mentioned. Nowadays, the LC of WPPB, produced mainly from polymer materials, generates the most problems [66,67].

The principal purpose of this inquiry was attained by acknowledgment of characterization of environmental consequences of the LC of selected post-production waste of WPPB.

The analyzes were construct on the Life Cycle Assessment method, and Eco-indicator 99 and Cumulative Energy Demand were employed as calculation procedures. The results were summarized separately for the eight selected waste from manufacturing processes of WPPB. Our study encloses nearly the total LC of the appraise post-production waste of WPPB (did not involve the periods of waste storage and transport to post-use management).

The conducted research allowed to determine the types of post-production waste from WPPB, which potentially pose the greatest threat to the environment. The proposed methodology provides an appropriate tool to identify the area of production processes that should be improved in terms of environmental protection. The obtained results contain clearly defined negative aspects of the LC of research objects, which should be mitigated or eliminated, e.g., by creating appropriate standards or legal acts.

The LC of resin discs made of epoxy resins was indicated by the soaring total volume of potential detrimental effect on the milieu (Figure 6). The production of epoxy resins entails plenty of energy and raw materials, which translates into a high level of environmental impact. 

The greatest number of potential negative influence on the milieu, in the case of all the inspected objects, was recorded for two impact categories: activity affiliated to the extraction of fossil fuels and inorganic compounds causing respiratory diseases (Table 1 and Table 2).

The mining industry and the processing of metals and fossil fuels are the causes of the greatest changes in the environment. Its functioning is based on the acquisition of various raw materials, and deriving out of the standpoint of milieu burden, the most important are the amounts of raw materials extracted and the methods of their extraction. Opencast methods are usually much more destructive compared to other methods of mining deposits. Treatment processes (for example, crushing, filtering, washing, metal reduction, enrichment, refining, etc.) also emit pollutants and generate waste. Pre-treatment of minerals, for which chemical solvents are usually used, also pose a significant menace to human health and the environment quality [68].

During the production of polymers, they are used, in conjunction with crude oil, coal and natural gas. The undertaking analogous to their extraction crucially lessens the environment quality. The elevated volume of developing adverse effects in this impact category was also documented for the resin discs lifecycle (Figure 17).

For inorganic compounds that induce respiratory diseases, the LC of roving fabric made of epoxy silane-coated fiberglass can cause the most potential harm to human health in this area (Figure 9). Emissions of NO_x_ and SO_2_ into the atmosphere were of key importance in the overall size of the potential negative impact in this area (Tables S5 and S6). 

Among the analyzed post-production waste of WPPB, the roving fabric and the fiberglass mat lifecycles were distinguished by the elevated volume of developing detrimental impact on human health. A significantly higher degree of destructive impact in this regard was noted of waste, which is mainly produced from fiberglass (Figure 17). The largest potential detrimental influence on the environment quality also has those wastes that contain the highest content of fiberglass, that is fiberglass mat and roving fabric. (Figure 19). However, when assessing all areas of influence, it is clear that these resources had the maximum share in the overall environmental impact of post-production waste of WPPB. The LC of resin discs was characterized by a massively elevated volume of harmful influence in this sector (Figure 22).

Among all the considered emission areas, emissions to the atmosphere constitute the largest share (Figure 19 and Figure 21). The undertaking of extraction of raw materials and production are usually related to the employ of energy procured from conventional sources, which increases the volume of potential obstructive influence on the milieu of the LC. The LC of resin disc contain the biggest energy-consuming differentiate to additional appraised waste, as a conclusion of which it was distinguished by the sizeable destructive influence on the milieu (Figure 23).

The employ of recycling processes could lessen the volume of detrimental impacts throughout the LC of a given type of WPPB, usually by ~30% (from ~18% for vacuum bag film to about 85% for distribution hoses) (Figure 6).

## 5. Conclusions

Renewable energy sources are now considered the future of global energy. Nowadays, increasingly more attention is being paid to meeting human needs, including energy needs, in the form of solutions that are as environmentally friendly as possible. WPPB are still the element that causes the most difficulties during post-use planning of WPP. The conducted research was aimed at conducting an ecological and energy life cycle analysis and evaluation, steering towards minimization and development (positive progress) of selected polymer waste produced during the manufacture of WPPB. The obtained results can form the basis for eco-design and, consequently, for the creation of new techniques for the production of WPPB, better in line with the assumptions of sustainable development.

Maintaining the current, irrational from the optimum of the economic–environment relationship, model of functioning consisting in the production and sale of products and the generation of waste and its depositing in the environment will not bring about a radical change in the level of environmental quality. This is called “open way of management”, consisting in the use of the resulting post-use resources to a small extent. The implementation of the new management principles for an extended interval of time would lead to a fundamental change in this model, it means to close the circulation of material and energy flows as much as possible: the closed-cycle economy. Realization of such a method of management would significantly increase the efficiency of using natural resources in production processes, while reducing the burden on the environment [69].

In the modern, “open” WPPB production system, the collected natural resources constitute the basis for the production of plastics, materials and elements, while the waste, pollutants and blades generated in production processes after exploitation, most often entirely constitute waste deposited in the environment. On the other hand, in a truly (partially) “closed” industrial system, both the waste generated in the processes and the blades after use (post-use resources) would be mostly recovered and returned to the same or another system. The degree of their use would be determined by the level of technology. Only waste that is not suitable for further use would be discharged and deposited into the environment. As a result, much smaller amounts of natural resources would be obtained from the environment than in an open system [70,71].

It is also possible to imagine a completely closed (albeit not entirely realistic now) system for the manufacture of WPPB, in which the raw materials supplied to it would remain in the aggregate. They would only undergo modifications in subsequent phases of the LC. The implementation of such a closed management system would require significant progress in the use of natural resources. A genuinely closed industrial system would function in a manner similar to that of natural ecosystems, in which, as a result of a long chain of change, only a small amount of non-recyclable waste remains in the environment. These small amounts of non-recyclable waste slow down the transformation of the environment, but without disturbing its balance and without harming ecosystems, which are also slowly changing. Striving to imitate the functioning of natural ecosystems in which the amount of non-recyclable final waste is minimized, leads directly to minimizing the consumption of environmental resources [72,73].

For this reason, it becomes necessary to change the techniques of manufacture, design, construction, exploitation and post-use management to more environmentally friendly. In spite of fact that formulating new or refining existing manufacture techniques of WPPB, singular recognition should be to reimburse the use of low-waste or non-waste technologies and to soaring the volume of recuperation and reuse of materials deploy in all technological processes, as well as generated waste. The consumption of natural resources should also be limited and substances with the lowest possible environmental toxicity should be used. This entails following both technological and scientific developments and increasing the energy and material efficiency of production processes. In this frame of reference, it is noteworthy crucial to identify the type of emissions to the environment of the generated pollutants and wastes, which will allow reducing their amount and impact range.

Bringing the production system of WPPB closer to the natural one would make the system more self-sufficient and closed. For this purpose, it is necessary to use natural resources more efficiently. The existing methods of blade production, which assumed the formation of pollutants and waste, and (to a large extent) their storage in the environment, should be increasingly replaced by activities that eliminate their formation. It will be possible to reach a largely self-sufficient and closed system when it is gradually replaced by a fully integrated model, in which industrial and service processes (related to use processes) will transform the circulating mass of materials from one form to another, with a certain their loss and energy consumption. Such a fully integrated management system should ensure the optimal use of natural resources along with the minimization of the amount of waste and pollution generated. This would be comparable to achieving a state of sustainable development in which there are rational economic-environmental relations [74,75,76,77].

## Figures and Tables

**Table 1:** Grouping and weighting outcome of milieu repercussions appearing in the LC of chosen post-production waste WPPB—Section 1 [Pt/1 Mg].

Impact Category	Fiberglass Mat		Roving Fabric		Resin Discs		Distribution Hoses	
	LC	R	LC	R	LC	R	LC	R
Carcinogens	30.03	3.31	4.00	3.14	0.03	3.31	3.12	1.94
Resp. organics	0.13	−0.46	0.16	−0.44	0.28	−0.46	0.59	−0.40
Resp. inorganics	51.16	−10.26	94.75	−9.75	44.33	−10.26	43.04	−30.94
Climate change	26.19	−1.81	45.26	−1.72	6.05	−1.81	13.26	−0.82
Radiation	0.50	<0.01	0.34	<0.01	<0.01	<0.01	0.05	<0.01
Ozone layer	0.02	−0.01	0.02	−0.01	<0.01	−0.01	<0.01	<0.01
Ecotoxicity	7.45	2.59	2.76	2.46	<0.01	2.59	0.80	2.57
Acidification/ Eutrophication	4.62	−2.49	7.67	−2.37	7.70	−2.49	4.91	−4.69
Land use	0.42	<0.01	1.29	<0.01	2.32	<0.01	2.40	<0.01
Minerals	0.48	<0.01	0.17	<0.01	0.02	<0.01	0.05	<0.01
Fossil fuels	186.21	−204.85	333.83	−194.61	812.31	−204.85	120.77	−127.77
**Total**	**307.20**	**−214.00**	**490.24**	**−203.30**	**873.04**	**−214.00**	**189.00**	**−160.11**

LC—life cycle, R—recycling.

**Table 2:** Grouping and weighting outcome of milieu repercussions appearing in the LC of chosen post-production waste WPPB—Section 2 [Pt/1 Mg].

Impact Category	Spiral Hoses with Resin		Vacuum Bag Film		Infusion Materials Residues		Surplus Materials	
	LC	R	LC	R	LC	R	LC	R
Carcinogens	1.21	2.85	2.28	0.33	3.41	2.94	0.82	3.26
Resp. organics	0.36	−0.44	0.13	−0.05	0.37	−0.44	0.32	−0.38
Resp. inorganics	44.65	−17.16	30.56	−1.03	48.42	−14.68	52.37	−11.32
Climate change	8.22	−1.48	9.94	−0.18	21.26	−1.53	12.25	−1.01
Radiation	0.14	<0.01	0.97	<0.01	0.31	0.00	0.05	<0.01
Ozone layer	1.12	−0.01	0.03	<0.01	4.81	−0.01	<0.01	−0.01
Ecotoxicity	0.58	2.58	2.54	0.26	1.24	2.53	0.48	2.54
Acidification/ Eutrophication	6.83	−3.22	2.74	−0.25	6.67	−2.93	6.78	−2.43
Land use	2.67	<0.01	4.02	<0.01	2.95	<0.01	2.48	<0.01
Minerals	0.01	<0.01	0.25	<0.01	0.03	<0.01	0.04	<0.01
Fossil fuels	607.98	−179.16	62.69	−20.49	532.80	−183.46	505.05	−196.54
**Total**	**673.78**	**−196.03**	**116.16**	**−21.40**	**622.28**	**−197.58**	**580.65**	**−205.89**

LC—life cycle, R—recycling

**Table 3:** The ramifications of grouping and weighting of milieu effects for the procedures associated to energy production for appointed post-production waste of elevators of WPPB—Section 1 [Pt/1 Mg].

Impact Category	Fiberglass Mat		Roving Fabric		Resin Discs		Distribution Hoses	
	LC	R	LC	R	LC	R	LC	R
Non renewable, fossil	250.78	−184.59	450.52	−175.36	874.33	−184.59	186.00	−114.24
Non-renewable, nuclear	37.59	69.97	65.34	66.47	<0.01	69.97	23.00	51.57
Renewable, biomass	1.27	<0.01	6.93	<0.01	<0.01	<0.01	0.13	<0.01
Renewable, wind, solar, geothe	0.64	<0.01	0.45	<0.01	<0.01	<0.01	0.06	<0.01
Renewable, water	4.26	9.39	4.86	8.92	<0.01	9.39	3.39	8.25
**Total**	**294.53**	**−105.23**	**528.10**	**−99.97**	**874.33**	**−105.23**	**212.58**	**−54.42**

LC—life cycle, R—recycling

**Table 4:** The ramifications of grouping and weighting of milieu effects for the procedures associated to energy production for appointed post-production waste of elevators of WPPB—Section 2 [Pt/1 Mg].

Impact Category	Spiral Hoses with Resin		Vacuum Bag Film		Infusion Materials Residues		Surplus Materials	
	LC	R	LC	R	LC	R	LC	R
Non renewable, fossil	670.82	−161.14	105.29	−18.46	602.41	−165.08	577.84	−175.92
Non-renewable, nuclear	11.55	63.84	49.84	7.00	16.24	64.50	11.76	69.96
Renewable, biomass	<0.01	<0.01	<0.01	<0.01	0.14	<0.01	1.11	<0.01
Renewable, wind, solar, geothe	<0.01	<0.01	<0.01	<0.01	0.03	<0.01	0.07	<0.01
Renewable, water	1.50	9.01	5.80	0.94	2.03	8.93	0.81	8.85
**Total**	**683.87**	**−88.29**	**160.93**	**−10.52**	**620.85**	**−91.65**	**591.59**	**−97.11**

LC—life cycle, R—recycling

## Data Availability

The data in the study are available on request from the corresponding author. The data are not publicly available due to privacy policies.

## Supplementary Materials

The following are available online at https://www.mdpi.com/article/10.3390/ma14174975/s1, Table S1: Grouping and weighting ramifications of milieu effects for carcinogenic compounds—Section 1 [Pt/1 Mg]. Table S2: Grouping and weighting ramifications of milieu effects for carcinogenic compounds—Section 2 [Pt/1 Mg]. Table S3: Grouping and weighting ramifications of milieu effects for organic compounds causing respiratory diseases—Section 1 [Pt/1 Mg]. Table S4: Grouping and weighting ramifications of milieu effects for organic compounds causing respiratory diseases—Section 2 [Pt/1 Mg]. Table S5: Grouping and weighting ramifications of milieu effects for inorganic compounds causing respiratory diseases—Section 1 [Pt/1 Mg]. Table S6: Grouping and weighting ramifications of milieu effects for inorganic compounds causing respiratory diseases—Section 2 [Pt/1 Mg]. Table S7: Grouping and weighting ramifications of milieu effects for compounds causing climate change—Section 1 [Pt/1 Mg]. Table S8: Grouping and weighting ramifications of milieu effects for compounds causing climate change—Section 2 [Pt/1 Mg]. Table S9: Grouping and weighting ramifications of milieu effects for radioactive substances—Section 1 [Pt/1 Mg]. Table S10: Grouping and weighting ramifications of milieu effects for radioactive substances—Section 2 [Pt/1 Mg]. Table S11: Grouping and weighting ramifications of milieu effects for compounds that expand the ozone hole—Section 1 [Pt/1 Mg]. Table S12: Grouping and weighting ramifications of milieu effects for compounds that expand the ozone hole—Section 2 [Pt/1 Mg]. Table S13: Grouping and weighting ramifications of milieu effects for ecotoxic compounds—Section 1 [Pt/1 Mg]. Table S14: Grouping and weighting ramifications of milieu effects for ecotoxic compounds—Section 2 [Pt/1 Mg]. Table S15: Grouping and weighting ramifications of milieu effects for compounds causing acidification or eutrophication—Section 1 [Pt/1 Mg]. Table S16: Grouping and weighting ramifications of milieu effects for compounds causing acidification or eutrophication—Section 2 [Pt/1 Mg]. Table S17: Grouping and weighting ramifications of milieu effects for land use processes—Section 1 [Pt/1 Mg]. Table S18: Grouping and weighting ramifications of milieu effects for land use processes—Section 2 [Pt/1 Mg]. Table S19: Grouping and weighting ramifications of milieu effects for undertaking analogous to the extraction of mineral resources—Section 1 [Pt/1 Mg]. Table S20: Grouping and weighting ramifications of milieu effects for undertaking analogous to the extraction of mineral resources—Section 2 [Pt/1 Mg]. Table S21: Grouping and weighting ramifications of milieu effects for undertaking analogous to the extraction of fossil fuels—Section 1 [Pt/1 Mg]. Table S22: Grouping and weighting ramifications of milieu effects for undertaking analogous to the extraction of fossil fuels—Section 2 [Pt/1 Mg]. Table S23: Grouping and weighting ramifications of milieu effects for substances harmful to human health—Section 1 [Pt/1 Mg]. Table S24: Grouping and weighting ramifications of milieu effects for substances harmful to human health—Section 2 [Pt/1 Mg]. Table S25: Grouping and weighting ramifications of milieu effects for substances and processes that diminish the environment quality—Section 1 [Pt/1 Mg]. Table S26: Grouping and weighting ramifications of milieu effects for substances and processes that reduce the environment quality—Section 2 [Pt/1 Mg]. Table S27: Grouping and weighting ramifications of milieu effects for undertaking analogous to the depletion of raw materials—Section 1 [Pt/1 Mg]. Table S28: Grouping and weighting ramifications of milieu effects for undertaking analogous to the depletion of raw materials—Section 2 [Pt/1 Mg].
