# Supplementary material for: Specification of Environmental Consequences of the Life Cycle of Selected Post-Production Waste of Wind Power Plants Blades
Source: Materials (Basel). 2021 Aug 31;14(17):4975. doi: 10.3390/ma14174975 (PMC8434586; doi:10.3390/ma14174975)
Supplement: Supplementary file 1 [file materials-14-04975-s001.zip › materials-1335516-SI.pdf]

Supplementary

Specification of environmental consequences of the life cycle of selected post-production waste of wind power plants blades

Katarzyna Piotrowska [1,*], Izabela Piasecka [2]

[1] Faculty of Mechanical Engineering, Lublin University of Technology, 20-618 Lublin, Poland; k.piotrowska@pollub.pl (K.P)
[2] Faculty of Mechanical Engineering, University of Science and Technology in Bydgoszcz, 85-796 Bydgoszcz, Poland; izabela.piasecka@utp.edu.pl (I.P)
* Correspondence: k.piotrowska@pollub.pl (K.P)

For all tables:
red highlight—the highest level of negative environmental consequences for a given unit,
green highlight—the highest level of positive environmental consequences for a given unit.

Table S1. Grouping and weighting ramifications of milieu effects for carcinogenic compounds – section 1 [Pt / 1 Mg].

SUBSTANCE	COMPARTMENT	FIBERGLASS MAT		ROVING FABRIC		RESIN DISCS		DISTRIBUTION HOSES	
		LIFE CYCLE	RECYCLING	LIFE CYCLE	RECYCLING	LIFE CYCLE	RECYCLING	LIFE CYCLE	RECYCLING
Arsenic	Air	8.56	x	0.03	x	x	x	0.86	x
Arsenic	Soil	0.31	x	x	x	x	x	0.03	x
Arsenic, ion	Water	3.65	1.91	2.90	1.82	< 0.01	1.91	0.37	0.50
Cadmium	Air	15.69	0.07	0.07	0.07	< 0.01	0.07	1.57	0.07
Cadmium	Soil	1.05	x	0.02	x	x	x	0.11	x
Cadmium, ion	Water	0.29	0.04	0.66	0.04	< 0.01	0.04	0.03	0.02
Ethane, 1,2-dichloro-	Air	x	x	0.01	x	x	x	x	x
Metallic ions, unspecified	Water	< 0.01	-0.18	< 0.01	-0.17	0.02	-0.18	0.07	-0.09
Metals, unspecified	Air	< 0.01	1.47	< 0.01	1.40	< 0.01	1.47	0.05	1.44
PAH, polycyclic aromatic hydrocarbons	Water	x	x	x	x	< 0.01	-0.01	x	x
Particulates, < 2.5 μm	Air	0.14	x	0.28	x	x	x	0.01	x
Propylene oxide	Air	0.06	x	x	x	x	x	0.01	x
Propylene oxide	Water	0.22	x	x	x	x	x	0.02	x
Remaining substances	x	0.06	-0.01	0.02	-0.01	< 0.01	< 0.01	0.01	< 0.01
**TOTAL**		**30.03**	**3.31**	**4.00**	**3.14**	**0.03**	**3.31**	**3.12**	**1.94**

Table S2. Grouping and weighting ramifications of milieu effects for carcinogenic compounds – section 2 [Pt / 1 Mg].

SUBSTANCE	COMPARTMENT	SPIRAL HOSES WITH RESIN		VACUUM BAG FILM		INFUSION MATERIALS RESIDUES		SURPLUS MATERIALS	
		LIFE CYCLE	RECYCLING	LIFE CYCLE	RECYCLING	LIFE CYCLE	RECYCLING	LIFE CYCLE	RECYCLING
Arsenic	Air	0.01	x	0.07	x	0.03	x	0.01	x
Arsenic, ion	Water	0.23	1.44	1.58	0.19	0.56	1.56	0.46	1.90
Cadmium	Air	0.12	0.07	0.50	0.01	0.25	0.07	0.01	0.07
Cadmium, ion	Water	0.02	0.03	0.08	< 0.01	0.07	0.04	0.11	0.04
Chloroform	Air	x	x	x	x	0.20	x	x	x
Chloroform	Water	0.03	x	x	x	0.06	x	x	x
Metallic ions, unspecified	Water	0.03	-0.15	0.03	-0.02	0.07	-0.15	0.14	-0.18
Metals, unspecified	Air	0.01	1.46	0.01	0.15	0.02	1.43	0.04	1.43
Methane, tetrachloro-, CFC-10	Air	0.74	x	x	x	2.10	x	x	x
Methane, tetrachloro-, CFC-10	Water	0.01	x	x	x	0.01	x	x	x
PAH, polycyclic aromatic hydrocarbons	Water	x	x	0.01	< 0.01	x	x	< 0.01	-0.01
Particulates, < 2.5 µm	Air	x	x	x	x	x	x	0.04	x
Remaining substances	x	0.01	< 0.01	< 0.01	< 0.01	0.03	< 0.01	0.01	< 0.01
**TOTAL**		**1.21**	**2.85**	**2.28**	**0.33**	**3.41**	**2.94**	**0.82**	**3.26**

Table S3. Grouping and weighting ramifications of milieu effects for organic compounds causing respiratory diseases – section 1 [Pt / 1 Mg].

SUBSTANCE	COMPARTMENT	FIBERGLASS MAT		ROVING FABRIC		RESIN DISCS		DISTRIBUTION HOSES	
		LIFE CYCLE	RECYCLING	LIFE CYCLE	RECYCLING	LIFE CYCLE	RECYCLING	LIFE CYCLE	RECYCLING
Benzene	Air	< 0.01	< 0.01	< 0.01	< 0.01	x	x	< 0.01	< 0.01
Hydrocarbons, aliphatic, alkanes, unspecified	Air	< 0.01	x	< 0.01	x	x	x	< 0.01	x
Hydrocarbons, aromatic	Air	< 0.01	< 0.01	< 0.01	< 0.01	< 0.01	< 0.01	< 0.01	< 0.01
Hydrocarbons, chlorinated	Air	x	x	x	x	< 0.01	< 0.01	< 0.01	-0,01
Hydrocarbons, unspecified	Air	< 0.01	< 0.01	< 0.01	< 0.01	0.28	< 0.01	0.57	< 0.01
Methane, fossil	Air	< 0.01	x	0.01	x	x	x	< 0.01	x
NMVOC, non-methane volatile organic compounds, unspecified origin	Air	0.10	-0.47	0.14	-0.44	< 0.01	-0.47	0.01	-0.40
Propene	Air	0.01	x	< 0.01	x	x	x	< 0.01	x
Remaining substances	x	< 0.01	< 0.01	< 0.01	< 0.01	< 0.01	< 0.01	< 0.01	< 0.01
**TOTAL**		**0.13**	**-0.46**	**0.16**	**-0.44**	**0.28**	**-0.46**	**0.59**	**-0.40**

Table S4. Grouping and weighting ramifications of milieu effects for organic compounds causing respiratory diseases – section 2 [Pt / 1 Mg].

SUBSTANCE	COMPARTMENT	SPIRAL HOSES WITH RESIN		VACUUM BAG FILM		INFUSION MATERIALS RESIDUES		SURPLUS MATERIALS	
		LIFE CYCLE	RECYCLING	LIFE CYCLE	RECYCLING	LIFE CYCLE	RECYCLING	LIFE CYCLE	RECYCLING
Aldehydes, unspecified	Air	< 0.01	< 0.01	x	x	< 0.01	< 0.01	< 0.01	< 0.01
Benzene	Air	< 0.01	< 0.01	x	x	< 0.01	< 0.01	< 0.01	< 0.01
Butane	Air	< 0.01	x	< 0.01	x	< 0.01	x	x	x
Ethene	Air	< 0.01	x	< 0.01	x	< 0.01	x	< 0.01	x
Hexane	Air	< 0.01	x	< 0.01	x	< 0.01	x	x	x
Hydrocarbons, aliphatic, alkanes, unspecified	Air	x	x	< 0.01	x	< 0.01	x	< 0.01	x
Hydrocarbons, aromatic	Air	< 0.01	< 0.01	x	x	< 0.01	< 0.01	< 0.01	< 0.01
Hydrocarbons, chlorinated	Air	< 0.01	< 0.01	x	x	< 0.01	< 0.01	< 0.01	< 0.01
Hydrocarbons, unspecified	Air	0.32	< 0.01	0.07	< 0.01	0.30	< 0.01	0.30	< 0.01
Methane	Air	< 0.01	< 0.01	< 0.01	< 0.01	< 0.01	< 0.01	< 0.01	< 0.01
Methane, dichloro-, HCC-30	Air	< 0.01	x	x	x	< 0.01	x	< 0.01	x
NMVOC, non-methane volatile organic compounds, unspecified origin	Air	0.03	-0.44	0.06	-0.05	0.06	-0.44	0.02	-0.38
Pentane	Air	< 0.01	x	< 0.01	x	< 0.01	x	x	x
Propene	Air	< 0.01	x	x	x	< 0.01	x	< 0.01	x
Xylene	Air	< 0.01	x	< 0.01	x	< 0.01	x	x	x
Remaining substances	x	< 0.01	< 0.01	< 0.01	< 0.01	< 0.01	< 0.01	< 0.01	< 0.01
**TOTAL**		**0.36**	**-0.44**	**0.13**	**-0.05**	**0.37**	**-0.44**	**0.32**	**-0.38**

Table S5. Grouping and weighting ramifications of milieu effects for inorganic compounds causing respiratory diseases – section 1 [Pt / 1 Mg].

SUBSTANCE	COMPARTMENT	FIBERGLASS MAT		ROVING FABRIC		RESIN DISCS		DISTRIBUTION HOSES	
		LIFE CYCLE	RECYCLING	LIFE CYCLE	RECYCLING	LIFE CYCLE	RECYCLING	LIFE CYCLE	RECYCLING
Ammonia	Air	0.22	0.01	0.08	0.01	x	x	0.02	0.01
Nitric oxide	Air	x	x	x	x	0.06	x	x	x
Nitrogen oxides	Air	18.48	-13.89	31.20	-13.20	39.19	-13.89	21.80	-23.25
Particulates	Air	< 0.01	0.40	< 0.01	0.38	< 0.01	0.40	7.48	-4.01
Particulates, < 2.5 µm	Air	9.87	x	19.93	x	x	x	0.99	x
Particulates, > 2.5 µm, and < 10 µm	Air	6.14	x	15.34	x	x	x	0.61	x
Particulates, SPM	Air	x	x	x	x	2.80	x	x	x
Sulfate	Air	1.18	x	x	x	x	x	0.12	x
Sulfur dioxide	Air	15.28	x	28.15	x	1.95	x	1.53	x
Sulfur oxides	Air	< 0.01	3.22	< 0.01	3.06	0.33	3.22	10.49	-3.69
Remaining substances	x	< 0.01	< 0.01	0.04	< 0.01	< 0.01	0.01	< 0.01	< 0.01
**TOTAL**		**51.16**	**-10.26**	**94.75**	**-9.75**	**44.33**	**-10.26**	**43.04**	**-30.94**

Table S6. Grouping and weighting ramifications of milieu effects for inorganic compounds causing respiratory diseases – section 2 [Pt / 1 Mg].

SUBSTANCE	COMPARTMENT	SPIRAL HOSES WITH RESIN		VACUUM BAG FILM		INFUSION MATERIALS RESIDUES		SURPLUS MATERIALS	
		LIFE CYCLE	RECYCLING	LIFE CYCLE	RECYCLING	LIFE CYCLE	RECYCLING	LIFE CYCLE	RECYCLING
Nitric oxide	Air	0.04	x	x	x	x	x	x	x
Nitrogen oxides	Air	33.39	-17.01	9.65	-1.39	31.19	-15.67	31.64	-12.96
Particulates	Air	1.66	-1.07	< 0.01	0.04	< 0.01	-0.59	< 0.01	0.39
Particulates, < 2.5 µm	Air	x	x	x	x	0.33	x	3.16	x
Particulates, > 2.5 µm, and < 10 µm	Air	x	x	x	x	0.13	x	2.43	x
Particulates, < 10 µm (mobile)	Air	0.03	x	0.10	x	0.06	x	x	x
Particulates, < 10 µm (stationary)	Air	0.95	x	4.68	x	1.94	x	x	x
Particulates, SPM	Air	1.96	x	0.86	x	3.40	x	3.52	x
Sulfate	Air	x	x	x	x	0.35	x	x	x
Sulfur dioxide	Air	1.36	x	x	x	2.47	x	5.34	x
Sulfur oxides	Air	5.25	0.92	15.27	0.32	8.51	1.57	6.22	1.24
Remaining substances	x	< 0.01	0.01	0.01	< 0.01	0.04	0.01	0.05	0.01
**TOTAL**		**44.65**	**-17.16**	**30.56**	**-1.03**	**48.42**	**-14.68**	**52.37**	**-11.32**

Table S7. Grouping and weighting ramifications of milieu effects for compounds causing climate change – section 1 [Pt / 1 Mg].

SUBSTANCE	COMPARTMENT	FIBERGLASS MAT		ROVING FABRIC		RESIN DISCS		DISTRIBUTION HOSES	
		LIFE CYCLE	RECYCLING	LIFE CYCLE	RECYCLING	LIFE CYCLE	RECYCLING	LIFE CYCLE	RECYCLING
Carbon dioxide	Air	< 0.01	-1.84	< 0.01	-1.75	6.03	-1.84	9.84	-0.69
Carbon dioxide, biogenic	Air	0.18	x	0.35	x	x	x	0.02	x
Carbon dioxide, fossil	Air	17.42	x	40.19	x	x	x	1.74	x
Carbon dioxide, in air	Raw	-0.17	x	-0.50	x	x	x	-0.02	x
Carbon monoxide, fossil	Air	0.03	x	0.06	x	x	x	x	x
Dinitrogen monoxide	Air	7.40	0.02	1.27	0.02	< 0.01	0.02	0.74	0.02
Methane	Air	x	x	x	x	< 0.01	0.01	0.78	-0.13
Methane, bromotrifluoro-, Halon 1301	Air	x	x	x	x	< 0.01	0.01	x	x
Methane, dichloro-, HCC-30	Air	x	x	x	x	0.02	x	x	x
Methane, fossil	Air	1.29	x	3.82	x	x	x	0.13	x
Remaining substances	x	0.03	0.01	0.07	0.01	< 0.01	< 0.01	0.02	-0.01
**TOTAL**		**26.19**	**-1.81**	**45.26**	**-1.72**	**6.05**	**-1.81**	**13.26**	**-0.82**

Table S8. Grouping and weighting ramifications of milieu effects for compounds causing climate change – section 2 [Pt / 1 Mg].

SUBSTANCE	COMPA-RTMENT	SPIRAL HOSES WITH RESIN		VACUUM BAG FILM		INFUSION MATERIALS RESIDUES		SURPLUS MATERIALS	
		LIFE CYCLE	RECY-CLING	LIFE CYCLE	RECY-CLING	LIFE CYCLE	RECY-CLING	LIFE CYCLE	RECY-CLING
Carbon dioxide	Air	8.15	-1.46	9.53	-0.18	8.19	-1.53	5.06	-1.08
Carbon dioxide, biogenic	Air	x	x	x	x	0.02	x	0.06	x
Carbon dioxide, fossil	Air	x	x	x	x	0.59	x	6.37	x
Carbon dioxide, in air	Raw	x	x	x	x	-0.02	x	-0.08	x
Dinitrogen monoxide	Air	0.01	0.02	0.09	< 0.01	0.03	0.02	0.20	0.02
Ethane, 1,1,1,2-tetrafluoro-, HFC-134a	Air	x	x	x	x	0.49	x	x	x
Methane	Air	0.27	-0.04	0.31	< 0.01	0.18	-0.02	< 0.01	0.04
Methane, bromotrifluoro-, Halon 1301	Air	x	x	-0.02	< 0.01	x	x	x	x
Methane, chlorodifluoro-, HCFC-22	Air	x	x	x	x	7.24	x	x	x
Methane, dichlorodifluoro-, CFC-12	Air	x	x	x	x	0.84	x	x	x
Methane, fossil	Air	x	x	x	x	0.03	x	0.61	x
Methane, tetrachloro-, CFC-10	Air	-0.23	x	x	x	-0.65	x	x	x
Methane, trifluoro-, HFC-23	Air	x	x	x	x	4.29	x	x	x
Remaining substances	x	0.01	< 0.01	0.02	< 0.01	0.02	< 0.01	0.03	0.01
**TOTAL**		**8.22**	**-1.48**	**9.94**	**-0.18**	**21.26**	**-1.53**	**12.25**	**-1.01**

Table S9. Grouping and weighting ramifications of milieu effects for radioactive substances – section 1 [Pt / 1 Mg].

SUBSTANCE	COMPA-RTMENT	FIBERGLASS MAT		ROVING FABRIC		RESIN DISCS		DISTRIBUTION HOSES	
		LIFE CYCLE	RECY-CLING	LIFE CYCLE	RECY-CLING	LIFE CYCLE	RECY-CLING	LIFE CYCLE	RECY-CLING
^{14}Carbon	Air	0.16	x	0.11	x	x	x	0.02	x
^{129}Iodine	Air	< 0.01	x	< 0.01	x	x	x	< 0.01	x
^{222}Radon	Air	0.33	x	0.23	x	x	x	0.03	x
Remaining substances	x	< 0.01	x	< 0.01	x	x	x	< 0.01	x
**TOTAL**		**0.50**	**x**	**0.34**	**x**	**x**	**x**	**0.05**	**x**

Table S10. Grouping and weighting ramifications of milieu effects for radioactive substances – section 2 [Pt / 1 Mg].

SUBSTANCE	COMPA-RTMENT	SPIRAL HOSES WITH RESIN		VACUUM BAG FILM		INFUSION MATERIALS RESIDUES		SURPLUS MATERIALS	
		LIFE CYCLE	RECY-CLING	LIFE CYCLE	RECY-CLING	LIFE CYCLE	RECY-CLING	LIFE CYCLE	RECY-CLING
^{14}Carbon	Air	0.01	x	0.08	x	0.03	x	0.02	x
^{134}Cesium	Water	< 0.01	x	< 0.01	x	< 0.01	x	x	x
^{137}Cesium	Water	0.01	x	0.05	x	0.01	x	x	x
^{60}Cobalt	Water	< 0.01	x	0.01	x	< 0.01	x	x	x
^{129}Iodine	Air	< 0.01	x	< 0.01	x	< 0.01	x	< 0.01	x
^{85}Krypton	Air	< 0.01	x	< 0.01	x	< 0.01	x	x	x
^{226}Radium	Water	< 0.01	x	< 0.01	x	< 0.01	x	x	x
^{222}Radon	Air	0.12	x	0.82	x	0.26	x	0.04	x
Remaining substances	x	< 0.01	x	< 0.01	x	< 0.01	x	< 0.01	x
**TOTAL**		**0.14**	**x**	**0.97**	**x**	**0.31**	**x**	**0.05**	**x**

Table S11. Grouping and weighting ramifications of milieu effects for compounds that expand the ozone hole – section 1 [Pt / 1 Mg].

SUBSTANCE	COMPA-RTMENT	FIBERGLASS MAT		ROVING FABRIC		RESIN DISCS		DISTRIBUTION HOSES	
		LIFE CYCLE	RECY-CLING	LIFE CYCLE	RECY-CLING	LIFE CYCLE	RECY-CLING	LIFE CYCLE	RECY-CLING
Ethane, 1,2-dichloro-1,1,2,2-tetrafluoro-, CFC-114	Air	< 0.01	x	< 0.01	x	x	x	< 0.01	x
Methane, bromochlorodifluoro-, Halon 1211	Air	< 0.01	x	< 0.01	x	x	x	< 0.01	x
Methane, bromotrifluoro-, Halon 1301	Air	< 0.01	-0.01	< 0.01	-0.01	< 0.01	-0.01	< 0.01	< 0.01
Methane, chlorodifluoro-, HCFC-22	Air	< 0.01	x	< 0.01	x	x	x	< 0.01	x
Methane, dichlorodifluoro-, CFC-12	Air	< 0.01	x	0.02	x	x	x	< 0.01	x
Methane, monochloro-, R-40	Air	< 0.01	x	< 0.01	x	x	x	< 0.01	x
Methane, tetrachloro-, CFC-10	Air	0.01	x	< 0.01	x	x	x	< 0.01	x
Remaining substances	x	< 0.01	< 0.01	< 0.01	< 0.01	< 0.01	< 0.01	< 0.01	< 0.01
**TOTAL**		**0.02**	**-0.01**	**0.02**	**-0.01**	< 0.01	**-0.01**	**< 0.01**	**< 0.01**

Table S12. Grouping and weighting ramifications of milieu effects for compounds that expand the ozone hole – section 2 [Pt / 1 Mg].

SUBSTANCE	COMPA-RTMENT	SPIRAL HOSES WITH RESIN		VACUUM BAG FILM		INFUSION MATERIALS RESIDUES		SURPLUS MATERIALS	
		LIFE CYCLE	RECY-CLING	LIFE CYCLE	RECY-CLING	LIFE CYCLE	RECY-CLING	LIFE CYCLE	RECY-CLING
Methane, bromotrifluoro-, Halon 1301	Air	0.01	-0.01	0.03	< 0.01	0.03	-0.01	< 0.01	-0,01
Methane, chlorodifluoro-, HCFC-22	Air	x	x	x	x	1.09	x	x	x
Methane, dichlorodifluoro-, CFC-12	Air	x	x	< 0.01	x	0.52	x	< 0.01	x
Methane, tetrachloro-, CFC-10	Air	1.11	x	< 0.01	x	3.16	x	< 0.01	x
Remaining substances	x	< 0.01	< 0.01	< 0.01	< 0.01	0.01	< 0.01	< 0.01	< 0.01
**TOTAL**		**1.12**	**-0.01**	**0.03**	**< 0.01**	**4.81**	**-0.01**	**< 0.01**	**-0.01**

Table S13. Grouping and weighting ramifications of milieu effects for ecotoxic compounds – section 1 [Pt / 1 Mg].

SUBSTANCE	COMPA-RTMENT	FIBERGLASS MAT		ROVING FABRIC		RESIN DISCS		DISTRIBUTION HOSES	
		LIFE CYCLE	RECY-CLING	LIFE CYCLE	RECY-CLING	LIFE CYCLE	RECY-CLING	LIFE CYCLE	RECY-CLING
Arsenic	Air	0.62	x	x	x	x	x	0.06	x
Cadmium	Air	3.36	0.01	0.02	0.01	< 0.01	0.01	0.34	0.02
Chromium	Air	0.56	x	0.34	x	x	x	0.06	x
Chromium	Water	< 0.01	0.03	< 0.01	0.03	< 0.01	0.03	< 0.01	0.01
Chromium, ion	Water	x	x	x	x	< 0.01	0.03	x	x
Chromium VI	Water	0.03	x	0.12	x	x	x	x	x
Copper	Air	0.11	x	0.02	x	x	x	0.01	x
Copper, ion	Water	0.08	0.03	0.46	0.03	x	x	0.01	0.01
Lead	Air	0.06	0.05	0.03	0.05	< 0.01	0.05	0.01	0.05
Mercury	Air	0.02	< 0.01	< 0.01	< 0.01	x	x	x	x
Metallic ions, unspecified	Water	< 0.01	1.61	x	x	< 0.01	< 0.01	x	x
Metals, unspecified	Air	x	x	< 0.01	1.53	< 0.01	1.61	0.05	1.58
Nickel	Air	2.24	0.78	1.31	0.74	< 0.01	0.78	0.22	0.85
Nickel, ion	Water	0.05	0.03	0.25	0.03	< 0.01	0.03	0.01	0.01
Zinc	Air	0.25	0.04	0.07	0.04	< 0.01	0.04	0.02	0.06
Zinc	Soil	0.05	x	0.05	x	x	x	< 0.01	x
Zinc, ion	Water	0.01	0.01	0.05	0.01	< 0.01	0.01	x	x
Remaining substances	x	0.02	< 0.01	0.03	< 0.01	< 0.01	< 0.01	0.01	< 0.01
**TOTAL**		**7.45**	**2.59**	**2.76**	**2.46**	**< 0.01**	**2.59**	**0.80**	**2.57**

Table S14. Grouping and weighting ramifications of milieu effects for ecotoxic compounds – section 2 [Pt / 1 Mg].

SUBSTANCE	COMPA-RTMENT	SPIRAL HOSES WITH RESIN		VACUUM BAG FILM		INFUSION MATERIALS RESIDUES		SURPLUS MATERIALS	
		LIFE CYCLE	RECY-CLING	LIFE CYCLE	RECY-CLING	LIFE CYCLE	RECY-CLING	LIFE CYCLE	RECY-CLING
Cadmium	Air	0.02	0.02	0.11	< 0.01	0.05	0.01	< 0.01	0.01
Chromium	Air	0.01	x	0.05	x	0.02	x	0.05	x
Chromium	Water	< 0.01	0.02	0.02	< 0.01	< 0.01	0.02	< 0.01	0.03
Chromium	Soil	0.01	x	0.02	x	0.02	x	x	x
Chromium VI	Water	x	x	x	x	x	x	0.02	x
Copper	Air	0.01	x	0.06	x	0.02	x	0.00	x
Copper, ion	Water	< 0.01	0.02	0.03	< 0.01	0.01	0.02	0.07	0.03
Lead	Air	0.02	0.05	0.10	0.01	0.04	0.05	0.01	0.05
Mercury	Air	< 0.01	< 0.01	< 0.01	< 0.01	0.01	< 0.01	x	x
Metals, unspecified	Air	0.01	1.60	0.01	0.16	0.02	1.57	0.04	1.57
Nickel	Air	0.43	0.80	1.89	0.08	0.90	0.78	0.21	0.78
Nickel, ion	Water	< 0.01	0.02	0.03	< 0.01	0.01	0.02	0.04	0.03
Zinc	Air	0.03	0.05	0.15	< 0.01	0.06	0.04	0.01	0.04
Zinc	Soil	0.02	x	0.05	x	0.05	x	0.01	x
Zinc, ion	Water	< 0.01	0.01	0.01	< 0.01	< 0.01	0.01	0.01	0.01
Remaining substances	x	< 0.01	< 0.01	0.02	< 0.01	0.02	< 0.01	0.01	< 0.01
**TOTAL**		**0.58**	**2.58**	**2.54**	**0.26**	**1.24**	**2.53**	**0.48**	**2.54**

Table S15. Grouping and weighting ramifications of milieu effects for compounds causing acidification or eutrophication – section 1 [Pt / 1 Mg].

SUBSTANCE	COMPA-RTMENT	FIBERGLASS MAT		ROVING FABRIC		RESIN DISCS		DISTRIBUTION HOSES	
		LIFE CYCLE	RECY-CLING	LIFE CYCLE	RECY-CLING	LIFE CYCLE	RECY-CLING	LIFE CYCLE	RECY-CLING
Ammonia	Air	0.12	< 0.01	0.04	< 0.01	x	x	0.01	< 0.01
Nitrogen oxides	Air	3.56	-2.68	6.02	-2.55	7.56	-2.68	4.21	-4.48
Sulfate	Air	0.07	x	x	x	x	x	0.01	x
Sulfur dioxide	Air	0.87	x	1.61	x	0.11	x	0.09	x
Sulfur oxides	Air	< 0.01	0.18	< 0.01	0.17	0.02	0.18	0.60	-0.21
Remaining substances	x	< 0.01	< 0.01	< 0.01	< 0.01	0.01	< 0.01	< 0.01	< 0.01
**TOTAL**		**4.62**	**-2.49**	**7.67**	**-2.37**	**7.70**	**-2.49**	**4.91**	**-4.69**

Table S16. Grouping and weighting ramifications of milieu effects for compounds causing acidification or eutrophication – section 2 [Pt / 1 Mg].

SUBSTANCE	COMPA-RTMENT	SPIRAL HOSES WITH RESIN		VACUUM BAG FILM		INFUSION MATERIALS RESIDUES		SURPLUS MATERIALS	
		LIFE CYCLE	RECY-CLING	LIFE CYCLE	RECY-CLING	LIFE CYCLE	RECY-CLING	LIFE CYCLE	RECY-CLING
Nitrogen oxides	Air	6.44	-3.28	1.86	-0.27	6.02	-3.02	6.11	-2,.0
Sulfate	Air	x	x	x	x	0.02	x	x	x
Sulfur dioxide	Air	0.08	x	x	x	0.14	x	0.30	x
Sulfur oxides	Air	0.30	0.05	0.87	0.02	0.49	0.09	0.36	0.07
Remaining substances	x	0.01	< 0.01	< 0.01	< 0.01	0.01	< 0.01	0.01	< 0.01
**TOTAL**		**6.83**	**-3.22**	**2.74**	**-0.25**	**6.67**	**-2.93**	**6.78**	**-2.43**

Table S17. Grouping and weighting ramifications of milieu effects for land use processes – section 1 [Pt / 1 Mg].

PROCESS	COMPA-RTMENT	FIBERGLASS MAT		ROVING FABRIC		RESIN DISCS		DISTRIBUTION HOSES	
		LIFE CYCLE	RECY-CLING	LIFE CYCLE	RECY-CLING	LIFE CYCLE	RECY-CLING	LIFE CYCLE	RECY-CLING
Occupation, arable, non-irrigated	Raw	< 0.01	x	0.01	x	x	x	x	x
Occupation, construction site	Raw	< 0.01	x	0.01	x	x	x	x	x
Occupation, dump site	Raw	0.12	x	0.09	x	x	x	0.01	x
Occupation, forest, intensive	Raw	< 0.01	x	0.01	x	x	x	x	x
Occupation, forest, intensive, normal	Raw	0.04	x	1.05	x	x	x	< 0.01	x
Occupation, industrial area	Raw	0.03	x	0.02	x	2.15	x	2.36	x
Occupation, mineral extraction site	Raw	0.23	x	0.05	x	x	x	0.02	x
Occupation, traffic area, road embankment	Raw	< 0.01	x	0.08	x	x	x	x	x
Transformation, from arable	Raw	-0.01	x	x	x	x	x	x	x
Transformation, from arable, non-irrigated	Raw	-0.03	x	-0.42	x	x	x	< 0.01	x
Transformation, from dump site, inert material landfill	Raw	-0.01	x	-0.03	x	x	x	x	x
Transformation, from dump site, residual material landfill	Raw	-0.01	x	-0.01	x	x	x	x	x
Transformation, from forest	Raw	-0.01	x	x	x	x	x	x	x
Transformation, from forest, extensive	Raw	-0.01	x	-0.26	x	x	x	x	x
Transformation, from mineral extraction site	Raw	-0.14	x	-0.03	x	x	x	-0.01	x
Transformation, from pasture and meadow	Raw	-0.03	x	-0.06	x	x	x	< 0.01	x
Transformation, from shrub land, sclerophyllous	Raw	< 0.01	x	-0.01	x	x	x	x	x
Transformation, from unknown	Raw	-0.32	x	-0.10	x	x	x	-0.03	x
Transformation, to arable	Raw	0.06	x	0.03	x	x	x	0.01	x
Transformation, to arable, non-irrigated	Raw	0.03	x	0.42	x	x	x	< 0.01	x
Transformation, to dump site	Raw	0.03	x	0.02	x	x	x	< 0.01	x
Transformation, to dump site, inert material landfill	Raw	0.01	x	0.03	x	x	x	x	x

Transformation, to dump site, residual material landfill	Raw	0.01	x	0.01	x	x	x	x	x
Transformation, to forest	Raw	0.01	x	0.01	x	x	x	x	x
Transformation, to forest, intensive, normal	Raw	0.01	x	0.26	x	x	x	x	x
Transformation, to industrial area	Raw	0.01	x	0.01	x	0.17	x	x	x
Transformation, to mineral extraction site	Raw	0.25	x	0.04	x	x	x	0.02	x
Transformation, to traffic area, road embankment	Raw	< 0.01	x	0.02	x	x	x	x	x
Transformation, to unknown	Raw	0.06	x	< 0.01	x	x	x	0.01	x
Transformation, to water bodies, artificial	Raw	0.06	x	0.03	x	x	x	0.01	x
Transformation, to water courses, artificial	Raw	0.02	x	0.02	x	x	x	x	x
Remaining substances	x	0.01	x	0.02	x	< 0.01	x	< 0.01	x
**TOTAL**		**0.42**	**x**	**1.29**	**x**	**2.32**	**x**	**2.40**	**x**

Table S18. Grouping and weighting ramifications of milieu effects for land use processes – section 2 [Pt / 1 Mg].

PROCESS	COMPARTMENT	SPIRAL HOSES WITH RESIN		VACUUM BAG FILM		INFUSION MATERIALS RESIDUES		SURPLUS MATERIALS	
		LIFE CYCLE	RECYCLING	LIFE CYCLE	RECYCLING	LIFE CYCLE	RECYCLING	LIFE CYCLE	RECYCLING
Land use II-III	Raw	0.52	x	3.57	x	1.04	x	x	x
Land use II-IV	Raw	< 0.01	x	0.10	x	0.01	x	x	x
Land use III-IV	Raw	x	x	0.07	x	0.01	x	x	x
Occupation, dump site	Raw	x	x	x	x	0.01	x	0.01	x
Occupation, forest, intensive, normal	Raw	x	x	x	x	0.01	x	0.17	x
Occupation, industrial area	Raw	2.03	x	0.27	x	1.78	x	2.20	x
Occupation, mineral extraction site	Raw	x	x	x	x	< 0.01	x	0.01	x
Transformation, from arable, non-irrigated	Raw	x	x	x	x	x	x	-0.07	x
Transformation, from forest, extensive	Raw	x	x	x	x	x	x	-0.04	x
Transformation, from pasture and meadow	Raw	x	x	x	x	x	x	-0.01	x
Transformation, from unknown	Raw	x	x	x	x	-0.01	x	-0.02	x
Transformation, to arable	Raw	x	x	x	x	x	x	0.01	x
Transformation, to arable, non-irrigated	Raw	x	x	x	x	x	x	0.07	x
Transformation, to forest, intensive, normal	Raw	x	x	x	x	x	x	0.04	x
Transformation, to industrial area	Raw	0.12	x	x	x	0.09	x	0.08	x
Transformation, to mineral extraction site	Raw	x	x	x	x	< 0.01	x	0.01	x
Remaining substances	x	< 0.01	x	< 0.01	x	0.01	x	0.02	x
**TOTAL**		**2.67**	**x**	**4.02**	**x**	**2.95**	**x**	**2.48**	**x**

Table S19. Grouping and weighting ramifications of milieu effects for undertaking analogous to the extraction of mineral resources – section 1 [Pt / 1 Mg].

PROCESS	COMPA-RTMENT	FIBERGLASS MAT		ROVING FABRIC		RESIN DISCS		DISTRIBUTION HOSES	
		LIFE CYCLE	RECY-CLING	LIFE CYCLE	RECY-CLING	LIFE CYCLE	RECY-CLING	LIFE CYCLE	RECY-CLING
Aluminium, 24% in bauxite, 11% in crude ore, in ground	Raw	0.46	x	0.03	x	x	x	0.05	x
Bauxite, in ground	Raw	< 0.01	< 0.01	< 0.01	< 0.01	0.02	< 0.01	< 0.01	< 0.01
Copper, 1.18% in sulfide, Cu 0.39% and Mo $8.2 \cdot 10^{-3}$% in crude ore, in ground	Raw	< 0.01	x	0.01	x	x	x	< 0.01	x
Copper, 2.19% in sulfide, Cu 1.83% and Mo $8.2 \cdot 10^{-3}$% in crude ore, in ground	Raw	< 0.01	x	0.02	x	x	x	< 0.01	x
Nickel, 1.98% in silicates, 1.04% in crude ore, in ground	Raw	0.01	x	0.03	x	x	x	< 0.01	x
Tin, 79% in cassiterite, 0.1% in crude ore, in ground	Raw	< 0.01	x	0.06	x	x	x	< 0.01	x
Remaining substances	x	< 0.01	< 0.01	< 0.01	< 0.01	< 0.01	< 0.01	< 0.01	< 0.01
**TOTAL**		**0.48**	**< 0.01**	**0.17**	**< 0.01**	**0.02**	**< 0.01**	**0.05**	**< 0.01**

Table S20. Grouping and weighting ramifications of milieu effects for undertaking analogous to the extraction of mineral resources – section 2 [Pt / 1 Mg].

PROCESS	COMPA-RTMENT	SPIRAL HOSES WITH RESIN		VACUUM BAG FILM		INFUSION MATERIALS RESIDUES		SURPLUS MATERIALS	
		LIFE CYCLE	RECY-CLING	LIFE CYCLE	RECY-CLING	LIFE CYCLE	RECY-CLING	LIFE CYCLE	RECY-CLING
Bauxite, in ground	Raw	0.01	< 0.01	0.01	< 0.01	0.01	< 0.01	0.01	< 0.01
Copper, in ground	Raw	x	x	0.22	x	< 0.01	x	x	x
Iron ore, in ground	Raw	< 0.01	< 0.01	x	x	< 0.01	< 0.01	0.01	< 0.01
Iron, in ground	Raw	< 0.01	x	0.01	x	< 0.01	x	x	x
Nickel, in ground	Raw	< 0.01	x	0.01	x	< 0.01	x	x	x
Tin, 79% in cassiterite, 0.1% in crude ore, in ground	Raw	x	x	x	x	0.01	x	0.01	x
Tin, in ground	Raw	x	x	0.01	x	< 0.01	x	x	x
Remaining substances	x	< 0.01	< 0.01	< 0.01	< 0.01	< 0.01	< 0.01	< 0.01	< 0.01
**TOTAL**		**0.01**	**< 0.01**	**0.25**	**< 0.01**	**0.03**	**< 0.01**	**0.04**	**< 0.01**

Table S21. Grouping and weighting ramifications of milieu effects for undertaking analogous to the extraction of fossil fuels – section 1 [Pt / 1 Mg].

PROCESS	COMPA-RTMENT	FIBERGLASS MAT		ROVING FABRIC		RESIN DISCS		DISTRIBUTION HOSES	
		LIFE CYCLE	RECY-CLING	LIFE CYCLE	RECY-CLING	LIFE CYCLE	RECY-CLING	LIFE CYCLE	RECY-CLING
Coal, 18 MJ per kg, in ground	Raw	< 0.01	1.23	< 0.01	1.17	< 0.01	1.23	< 0.01	0.54
Coal, hard, unspecified, in ground	Raw	1.28	x	3.33	x	x	x	0.13	x
Gas, mine, off-gas, process, coal mining/m³	Raw	0.37	x	0.21	x	x	x	0.04	x
Gas, natural, 30.3 MJ per kg, in ground	Raw	x	x	x	x	75.60	x	48.73	x
Gas, natural, 35 MJ per m³, in ground	Raw	< 0.01	10.06	< 0.01	9.56	< 0.01	10.06	< 0.01	10.06
Gas, natural, 36.6 MJ per m³, in ground	Raw	< 0.01	-63.50	< 0.01	-60.33	< 0.01	-63.50	< 0.01	-48.68
Gas, natural, feedstock, 35 MJ per m³, in ground	Raw	< 0.01	-69.72	< 0.01	-66.24	< 0.01	-69.72	< 0.01	-40.37
Gas, natural, in ground	Raw	109.51	x	209.73	x	x	x	10.95	x
Oil, crude, 42.6 MJ per kg, in ground	Raw	< 0.01	-17.20	< 0.01	-16.34	< 0.01	-17.20	< 0.01	-0.66
Oil, crude, 42.7 MJ per kg, in ground	Raw	x	x	x	x	736.71	x	52.69	x
Oil, crude, feedstock, 41 MJ per kg, in ground	Raw	< 0.01	-65.72	< 0.01	-62.43	< 0.01	-65.72	< 0.01	-48.66
Oil, crude, in ground	Raw	75.05	x	120.57	x	x	x	7.50	x
Remaining substances	x	< 0.01	< 0.01	< 0.01	< 0.01	< 0.01	< 0.01	0.74	< 0.01
**TOTAL**		**186.21**	**-204.85**	**333.83**	**-194.61**	**812.31**	**-204.85**	**120.77**	**-127.77**

Table S22. Grouping and weighting ramifications of milieu effects for undertaking analogous to the extraction of fossil fuels – section 2 [Pt / 1 Mg].

PROCESS	COMPA-RTMENT	SPIRAL HOSES WITH RESIN		VACUUM BAG FILM		INFUSION MATERIALS RESIDUES		SURPLUS MATERIALS	
		LIFE CYCLE	RECY-CLING	LIFE CYCLE	RECY-CLING	LIFE CYCLE	RECY-CLING	LIFE CYCLE	RECY-CLING
Coal, 18 MJ per kg, in ground	Raw	0.15	1.00	1.03	0.12	0.30	1.05	< 0.01	1.27
Gas, mine, off-gas, process, coal mining/m3	Raw	x	x	0.29	x	x	x	x	x
Gas, natural, 30.3 MJ per kg, in ground	Raw	63.75	x	9.26	x	61.46	x	51.47	x
Gas, natural, 35 MJ per m³, in ground	Raw	0.93	10.06	5.98	1.01	1.87	9.86	< 0.01	9.96
Gas, natural, 36.6 MJ per m³, in ground	Raw	< 0.01	-58.56	< 0.01	-6.35	< 0.01	-58.67	< 0.01	-52.59
Gas, natural, feedstock, 35 MJ per m³, in ground	Raw	< 0.01	-59.94	< 0.01	-6.97	< 0.01	-61.39	< 0.01	-53.21
Gas, natural, in ground	Raw	x	x	< 0.01	x	4.03	x	33.31	x
Oil, crude, 42.6 MJ per kg, in ground	Raw	15.54	-11.69	33.88	-1.72	31.32	-13.32	< 0.01	-22.02
Oil, crude, 42.7 MJ per kg, in ground	Raw	527.41	x	12.18	x	432.55	x	400.44	x
Oil, crude, feedstock, 41 MJ per kg, in ground	Raw	< 0.01	-60.03	< 0.01	-6.57	< 0.01	-61.00	< 0.01	-79.95
Oil, crude, in ground	Raw	x	x	x	x	0.98	x	19.13	x
Remaining substances	x	0.20	< 0.01	0.07	< 0.01	0.29	< 0.01	0.71	< 0.01

TOTAL	607.98	-179.16	62.69	-20.49	532.80	-183.46	505.05	-196.54

Table S23. Grouping and weighting ramifications of milieu effects for substances harmful to human health – section 1 [Pt / 1 Mg].

SUBSTANCE	COMPARTMENT	FIBERGLASS MAT		ROVING FABRIC		RESIN DISCS		DISTRIBUTION HOSES	
		LIFE CYCLE	RECYCLING	LIFE CYCLE	RECYCLING	LIFE CYCLE	RECYCLING	LIFE CYCLE	RECYCLING
Ammonia	Air	0.22	0.01	x	x	x	x	x	x
Arsenic	Air	8.56	x	x	x	x	x	0.86	x
Arsenic	Soil	0.31	x	x	x	x	x	0.03	x
Arsenic, ion	Water	3.65	1.91	2.90	1.82	< 0.01	1.91	0.37	0.50
Cadmium	Air	15.69	0.07	0.07	0.07	< 0.01	0.07	1.57	0.07
Cadmium	Soil	1.05	x	x	x	x	x	0.11	x
Cadmium, ion	Water	0.29	0.04	0.66	0.04	< 0.01	0.04	0.03	0.02
^{14}Carbon	Air	0.16	x	x	x	x	x	x	x
Carbon dioxide	Air	< 0.01	-1.84	< 0.01	-1.75	6.03	-1.84	9.84	-0.69
Carbon dioxide, biogenic	Air	0.18	x	0.35	x	x	x	x	x
Carbon dioxide, fossil	Air	17.42	x	40.19	x	x	x	1.74	x
Carbon dioxide, in air	Raw	-0.17	x	-0.50	x	x	x	x	x
Dinitrogen monoxide	Air	7.40	0.02	1.27	0.02	x	x	0.74	0.02
Hydrocarbons, unspecified	Air	x	x	x	x	0.28	< 0.01	0.57	< 0.01
Metallic ions, unspecified	Water	< 0.01	-0.18	< 0.01	-0.17	0.02	-0.18	x	x
Metals, unspecified	Air	< 0.01	1.47	< 0.01	1.40	< 0.01	1.47	0.05	1.44
Methane	Air	x	x	x	x	x	x	0.79	-0.13
Methane, fossil	Air	1.30	x	3.84	x	x	x	0.13	x
Nitric oxide	Air	x	x	x	x	0.06	x	x	x
Nitrogen oxides	Air	18.48	-13.89	31.20	-13.20	39.19	-13.89	21.80	-23.25
NMVOC, non-methane volatile organic compounds, unspecified origin	Air	0.10	-0.47	0.14	-0.44	< 0.01	-0.47	0.01	-0.40
Particulates	Air	< 0.01	0.40	< 0.01	0.38	< 0.01	0.40	7.48	-4.01
Particulates, < 2.5 µm	Air	10.00	x	20.21	x	x	x	1.00	x
Particulates, > 2.5 µm, and < 10 µm	Air	6.14	x	15.34	x	x	x	0.61	x
Particulates, SPM	Air	x	x	x	x	2.80	x	x	x
Propylene oxide	Water	0.22	x	0.23	x	x	x	x	x
^{222}Radon	Air	0.33	x	x	x	x	x	0.03	x
Sulfate	Air	1.18	x	x	x	x	x	0.12	x
Sulfur dioxide	Air	15.28	x	28.15	x	1.95	x	1.53	x
Sulfur oxides	Air	< 0.01	3.22	< 0.01	3.06	0.33	3.22	10.49	-3.69
Remaining substances	x	0.23	< 0.01	0.46	0.01	0.03	0.02	0.17	-0.10
**TOTAL**		**108.02**	**-9.24**	**144.53**	**-8.78**	**50.69**	**-9.24**	**60.06**	**-30.21**

Table S24. Grouping and weighting ramifications of milieu effects for substances harmful to human health – section 2 [Pt / 1 Mg].

SUBSTANCE	COMPA-RTMENT	SPIRAL HOSES WITH RESIN		VACUUM BAG FILM		INFUSION MATERIALS RESIDUES		SURPLUS MATERIALS	
		LIFE CYCLE	RECY-CLING	LIFE CYCLE	RECY-CLING	LIFE CYCLE	RECY-CLING	LIFE CYCLE	RECY-CLING
Arsenic, ion	Water	0.23	1.44	1.58	0.19	0.56	1.56	0.46	1.90
Cadmium	Air	0.12	0.07	0.50	0.01	0.25	0.07	0.01	0.07
Cadmium, ion	Water	0.02	0.03	0.08	< 0.01	0.07	0.04	0.11	0.04
Carbon dioxide	Air	8.15	-1.46	9.53	-0.18	8.19	-1.53	5.06	-1.08
Carbon dioxide, fossil	Air	x	x	x	x	0.59	x	6.37	x
Carbon dioxide, in air	Raw	x	x	x	x	x	x	-0.08	x
Chloroform	Air	x	x	x	x	0.21	x	x	x
Dinitrogen monoxide	Air	x	x	0.09	< 0.01	x	x	0.20	0.02
Ethane, 1,1,1,2-tetrafluoro-, HFC-134a	Air	x	x	x	x	0.49	x	x	x
Hydrocarbons, unspecified	Air	0.32	< 0.01	0.07	< 0.01	0.30	< 0.01	0.30	< 0.01
Metallic ions, unspecified	Water	0.03	-0.15	x	x	0.07	-0.15	x	x
Metals, unspecified	Air	0.01	1.46	0.01	0.15	0.02	1.43	0.04	1.43
Methane	Air	0.27	-0.04	0.31	< 0.01	0.18	-0.02	x	x
Methane, fossil	Air	x	x	x	x	x	x	0.61	x
Methane, chlorodifluoro-, HCFC-22	Air	x	x	x	x	8.33	x	x	x
Methane, dichlorodifluoro-, CFC-12	Air	x	x	x	x	1.36	x	x	x
Methane, tetrachloro-, CFC-10	Air	1.61	x	x	x	4.62	x	x	x
Methane, trifluoro-, HFC-23	Air	x	x	x	x	4.29	x	x	x
Nitrogen oxides	Air	33.39	-17.01	9.65	-1.39	31.19	-15.67	31.64	-12.96
NMVOC, non-methane volatile organic compounds, unspecified origin	Air	0.03	-0.44	x	x	0.06	-0.44	0.02	-0.38
Particulates	Air	1.66	-1.07	x	x	< 0.01	-0.59	< 0.01	0.39
Particulates, < 2.5 μm	Air	x	x	x	x	0.34	x	3.21	x
Particulates, > 2.5 μm, and < 10 μm	Air	x	x	x	x	0.13	x	2.43	x
Particulates, < 10 μm (mobile)	Air	x	x	0.10	x	x	x	x	x
Particulates, < 10 μm (stationary)	Air	0.95	x	4.68	x	1.94	x	x	x
Particulates, SPM	Air	1.96	x	0.86	x	3.40	x	3.52	x
^{222}Radon	Air	0.12	x	0.82	x	0.26	x	x	x
Sulfate	Air	x	x	x	x	0.35	x	x	x
Sulfur dioxide	Air	1.36	x	x	x	2.47	x	5.34	x
Sulfur oxides	Air	5.25	0.92	15.27	0.32	8.51	1.57	6.22	1.24
Remaining substances	x	0.21	0.01	0.37	-0.02	0.40	0.01	0.35	-0.14
**TOTAL**		**55.70**	**-16.23**	**43.92**	**-0.92**	**78.58**	**-13.72**	**65.81**	**-9.46**

Table S25. Grouping and weighting ramifications of milieu effects for substances and processes that diminish the environment quality – section 1 [Pt / 1 Mg].

PROCESS OR SUBSTANCE	COMPARTMENT	FIBERGLASS MAT		ROVING FABRIC		RESIN DISCS		DISTRIBUTION HOSES	
		LIFE CYCLE	RECYCLING	LIFE CYCLE	RECYCLING	LIFE CYCLE	RECYCLING	LIFE CYCLE	RECYCLING
Ammonia	Air	0.12	< 0.01	0.04	< 0.01	x	x	0.01	< 0.01
Arsenic	Air	0.62	x	x	x	x	x	0.06	x
Cadmium	Air	3.36	0.01	0.02	0.01	< 0.01	0.01	0.34	0.02
Chromium	Air	0.56	x	0.34	x	x	x	0.06	x
Chromium	Water	< 0.01	0.03	< 0.01	0.03	< 0.01	0.03	< 0.01	0.01
Chromium VI	Water	0.03	x	0.12	x	x	x	x	x
Copper	Air	0.11	x	0.02	x	x	x	0.01	x
Copper, ion	Water	0.08	0.03	0.46	0.03	< 0.01	0.03	0.01	0.01
Lead	Air	0.06	0.05	0.03	0.05	< 0.01	0.05	0.01	0.05
Metals, unspecified	Air	< 0.01	1.61	< 0.01	1.53	< 0.01	1.61	0.05	1.58
Nickel	Air	2.24	0.78	1.31	0.74	< 0.01	0.78	0.22	0.85
Nickel, ion	Water	0.05	0.03	0.25	0.03	< 0.01	0.03	0.01	0.01
Nitrogen oxides	Air	3.56	-2.68	6.02	-2.55	7.56	-2.68	4.21	-4.48
Occupation, dump site	Raw	0.12	x	0.09	x	x	x	0.01	x
Occupation, forest, intensive, normal	Raw	0.04	x	1.05	x	x	x	x	x
Occupation, industrial area	Raw	0.03	x	0.02	x	2.15	x	2.36	x
Occupation, mineral extraction site	Raw	0.23	x	0.05	x	x	x	0.02	x
Sulfate	Air	0.07	x	x	x	x	x	0.01	x
Sulfur dioxide	Air	0.87	x	1.61	x	0.11	x	0.09	x
Sulfur oxides	Air	< 0.01	0.18	< 0.01	0.17	0.02	0.18	0.60	-0.21
Transformation, from arable, non-irrigated	Raw	-0.03	x	-0.42	x	x	x	x	x
Transformation, from forest, extensive	Raw	x	x	-0.26	x	x	x	x	x
Transformation, from mineral extraction site	Raw	-0.14	x	-0.03	x	x	x	-0.01	x
Transformation, from pasture and meadow	Raw	-0.03	x	-0.06	x	x	x	x	x
Transformation, from unknown	Raw	-0.32	x	-0.10	x	x	x	-0.03	x
Transformation, to arable	Raw	0.06	x	0.03	x	x	x	0.01	x
Transformation, to arable, non-irrigated	Raw	0.03	x	0.42	x	x	x	x	x
Transformation, to forest, intensive, normal	Raw	x	x	0.26	x	x	x	x	x
Transformation, to industrial area	Raw	x	x	x	x	0.17	x	x	x
Transformation, to mineral extraction site	Raw	0.25	x	0.04	x	x	x	0.02	x
Transformation, to water bodies, artificial	Raw	0.06	x	0.03	x	x	x	0.01	x
Zinc	Air	0.25	0.04	0.07	0.04	< 0.01	0.04	0.02	0.06
Zinc	Soil	0.05	x	0.05	x	x	x	x	x
Zinc, ion	Water	0.01	0.01	0.05	0.01	x	x	x	x
Remaining substances	x	0.16	< 0.01	0.22	< 0.01	0.01	0.01	0.03	< 0.01
**TOTAL**		**12.49**	**0.10**	**11.72**	**0.09**	**10.03**	**0.10**	**8.11**	**-2.12**

Table S26. Grouping and weighting ramifications of milieu effects for substances and processes that reduce the environment quality – section 2 [Pt / 1 Mg].

PROCESS OR SUBSTANCE	COMPARTMENT	SPIRAL HOSES WITH RESIN		VACUUM BAG FILM		INFUSION MATERIALS RESIDUES		SURPLUS MATERIALS	
		LIFE CYCLE	RECYCLING	LIFE CYCLE	RECYCLING	LIFE CYCLE	RECYCLING	LIFE CYCLE	RECYCLING
Cadmium	Air	0.02	0.02	0.11	< 0.01	0.05	0.01	< 0.01	0.01
Chromium	Air	0.01	x	0.05	x	0.02	x	0.05	x
Chromium	Water	< 0.01	0.02	0.02	< 0.01	0.00	0.02	< 0.01	0.03
Chromium	Soil	0.01	x	0.02	x	0.02	x	x	x
Copper	Air	0.01	x	0.06	x	0.02	x	x	x
Copper, ion	Water	< 0.01	0.02	0.03	< 0.01	0.01	0.02	0.07	0.03
Land use II-III	Raw	0.52	x	3.57	x	1.04	x	x	x
Land use II-IV	Raw	x	x	0.10	x	x	x	x	x
Lead	Air	0.02	0.05	0.10	0.01	0.04	0.05	0.01	0.05
Metals, unspecified	Air	0.01	1.60	0.01	0.16	0.02	1.57	0.04	1.57
Nickel	Air	0.43	0.80	1.89	0.08	0.90	0.78	0.21	0.78
Nickel, ion	Water	< 0.01	0.02	0.03	< 0.01	0.01	0.02	0.04	0.03
Nitric oxide	Air	x	x	1.86	-0.27	x	x	x	x
Nitrogen oxides	Air	6.44	-3.28	x	x	6.02	-3.02	6.11	-2.50
Occupation, forest, intensive, normal	Raw	x	x	x	x	0.01	x	0.17	x
Occupation, industrial area	Raw	2.03	x	0.27	x	1.78	x	2.20	x
Sulfur dioxide	Air	0.08	x	x	x	0.14	x	0.30	x
Sulfur oxides	Air	0.30	0.05	0.87	0.02	0.49	0.09	0.36	0.07
Transformation, from arable, non-irrigated	Raw	x	x	x	x	x	x	-0.07	x
Transformation, from forest, extensive	Raw	x	x	x	x	x	x	-0.04	x
Transformation, to arable, non-irrigated	Raw	x	x	x	x	x	x	0.07	x
Transformation, to industrial area	Raw	0.12	x	x	x	0.09	x	0.08	x
Zinc	Air	0.03	0.05	0.15	< 0.01	0.06	0.04	0.01	0.04
Zinc	Soil	0.02	x	0.05	x	0.05	x	x	x
Remaining substances	x	0.03	0.01	0.10	< 0.01	0.08	0.01	0.13	0.01
**TOTAL**		**10.08**	**-0.64**	**9.30**	**0.01**	**10.86**	**-0.40**	**9.74**	**0.12**

Table S27. Grouping and weighting ramifications of milieu effects for undertaking analogous to the depletion of raw materials – section 1 [Pt / 1 Mg].

PROCESS	COMPA-RTMENT	FIBERGLASS MAT		ROVING FABRIC		RESIN DISCS		DISTRIBUTION HOSES	
		LIFE CYCLE	RECY-CLING	LIFE CYCLE	RECY-CLING	LIFE CYCLE	RECY-CLING	LIFE CYCLE	RECY-CLING
Aluminium, 24% in bauxite, 11% in crude ore, in ground	Raw	0.46	x	x	x	x	x	0.05	x
Coal, 18 MJ per kg, in ground	Raw	< 0.01	1.23	< 0.01	1.17	< 0.01	1.23	< 0.01	0.54
Coal, 29.3 MJ per kg, in ground	Raw	x	x	x	x	x	x	0.73	x
Coal, hard, unspecified, in ground	Raw	1.28	x	3.33	x	x	x	0.13	x
Gas, mine, off-gas, process, coal mining/m^3	Raw	0.37	x	0.21	x	x	x	0.04	x
Gas, natural, 30.3 MJ per kg, in ground	Raw	x	x	x	x	75.60	x	48.73	x
Gas, natural, 35 MJ per m^3, in ground	Raw	< 0.01	10.06	< 0.01	9.56	< 0.01	10.06	< 0.01	10.06
Gas, natural, 36.6 MJ per m^3, in ground	Raw	< 0.01	-63.50	< 0.01	-60.33	< 0.01	-63.50	< 0.01	-48.68
Gas, natural, feedstock, 35 MJ per m^3, in ground	Raw	< 0.01	-69.72	< 0.01	-66.24	< 0.01	-69.72	< 0.01	-40.37
Gas, natural, in ground	Raw	109.51	x	209.73	x	x	x	10.95	x
Oil, crude, 42.6 MJ per kg, in ground	Raw	< 0.01	-17.20	< 0.01	-16.34	< 0.01	-17.20	< 0.01	-0.66
Oil, crude, 42.7 MJ per kg, in ground	Raw	x	x	x	x	736.71	x	52.69	x
Oil, crude, feedstock, 41 MJ per kg, in ground	Raw	< 0.01	-65.72	< 0.01	-62.43	< 0.01	-65.72	< 0.01	-48.66
Oil, crude, in ground	Raw	75.05	x	120.57	x	x	x	7.50	x
Remaining substances	x	0.02	< 0.01	0.17	< 0.01	0.02	< 0.01	0.01	< 0.01
**TOTAL**		**186.69**	**-204.86**	**334.00**	**-194.61**	**812.33**	**-204.86**	**120.83**	**-127.78**

Table S28. Grouping and weighting ramifications of milieu effects for undertaking analogous to the depletion of raw materials – section 2 [Pt / 1 Mg].

PROCESS	COMPA-RTMENT	SPIRAL HOSES WITH RESIN		VACUUM BAG FILM		INFUSION MATERIALS RESIDUES		SURPLUS MATERIALS	
		LIFE CYCLE	RECY-CLING	LIFE CYCLE	RECY-CLING	LIFE CYCLE	RECY-CLING	LIFE CYCLE	RECY-CLING
Coal, 18 MJ per kg, in ground	Raw	0.15	1.00	1.03	0.12	0.30	1.05	< 0.01	1.27
Coal, hard, unspecified, in ground	Raw	x	x	x	x	x	x	0.53	x
Copper, in ground	Raw	x	x	0.22	x	x	x	x	x
Gas, mine, off-gas, process, coal mining/m³	Raw	x	x	0.29	x	x	x	x	x
Gas, natural, 30.3 MJ per kg, in ground	Raw	63.75	x	9.26	x	61.46	x	51.47	x
Gas, natural, 35 MJ per m³, in ground	Raw	0.93	10.06	5.98	1.01	1.87	9.86	< 0.01	9.96
Gas, natural, 36.6 MJ per m³, in ground	Raw	< 0.01	-58.56	< 0.01	-6.35	< 0.01	-58.67	< 0.01	-52.59
Gas, natural, feedstock, 35 MJ per m³, in ground	Raw	< 0.01	-59.94	< 0.01	-6.97	< 0.01	-61.39	< 0.01	-53.21
Gas, natural, in ground	Raw	x	x	x	x	4.03	x	33.31	x
Oil, crude, 42.6 MJ per kg, in ground	Raw	15.54	-11.69	33.88	-1.72	31.32	-13.32	< 0.01	-22.02
Oil, crude, 42.7 MJ per kg, in ground	Raw	527.41	x	12.18	x	432.55	x	400.44	x
Oil, crude, feedstock, 41 MJ per kg, in ground	Raw	< 0.01	-60.03	< 0.01	-6.57	< 0.01	-61.00	< 0.01	-79.95
Oil, crude, in ground	Raw	x	x	x	x	0.98	x	19.13	x
Remaining substances	x	0.22	< 0.01	0.10	< 0.01	0.33	< 0.01	0.21	< 0.01
**TOTAL**		**608.00**	**-179.16**	**62.94**	**-20.49**	**532.84**	**-183.46**	**505.09**	**-196.55**
